# Superdirective dimers of coupled self-resonant split ring resonators: Analytical modelling and numerical and experimental validation

**DOI:** 10.1038/s41598-019-56988-6

**Published:** 2020-01-14

**Authors:** A. Vallecchi, A. Radkovskaya, L. Li, G. Faulkner, C. J. Stevens, E. Shamonina

**Affiliations:** 1 Department of Engineering Science, University of Oxford, Parks Road, Oxford, OX1 3PJ United Kingdom; 2 Magnetism Division, Faculty of Physics, M. V. Lomonosov Moscow State University, Leninskie Gory, Moscow, 119992 Russia; 3 Present Address: Insight Lifetech, Shenzhen, 518052 P.R. China

**Keywords:** Electrical and electronic engineering, Computational science, Electronic and spintronic devices

## Abstract

Superdirective antennas developed over the last century have received renewed interest in recent years from the development of metamaterials. These arrays of electromagnetic resonators (or meta-atoms) carrying short wavelength electro- and/or magneto-inductive waves support current distributions with very high spatial frequency as required by the classical conditions for superdirectivity. As meta-atoms can have both electric and magnetic dipole characteristics (and hence radiation properties), developing antennas exploiting these distributions can challenge conventional intuitions regarding the optimal configurations required. In this work we are reporting the development of a genuinely superdirective array using split ring resonators (SRRs). We provide a comprehensive analytical model characterizing the radiation from SRR dimers in which excitation of only one split ring leads to superdirective radiation via mutually coupled modes. Our model exploits simple circuit descriptions of coupled resonant circuits, combined with standard radiation formulae for curvilinear current distributions. Using this simple model we are able to map directivity against possible SRR locations and orientations in two dimensions and identify the unique optimal configuration which meets the requirements for superdirective emission. We validate the theoretical findings by comparison to both full wave simulations and experiments showing that our SRR dimer achieves endfire directivity very close to the maximum theoretical value.

## Introduction

Directional and smart antennas are one of the approaches that could be used to increase the capacity and flexibility of wireless systems in future integrated gigabit communications (5G and beyond). The advantages of a steerable antenna system include the possibility of tracking a terminal signal and mitigating interference between network nodes, thus improving network capacity. Directional antennas can also improve power efficiency^1^. The most common technique to realize a directive antenna consists in assembling a multiple-antenna *array* in which the radiation pattern can be reinforced in a particular direction and suppressed in undesired directions. By individually controlling each array element in phase and amplitude, the direction of radiation can be electronically steered (*phased arrays*)^2^. Multi-element arrays are by now widely used in based stations of cellular networks, but simple implementation for mobile devices is severely constrained by the need for small antennas, the conventional phased array techniques for enhancing the directivity leading to a significant increase of the antenna size and requiring a separate RF chain for each antenna element. On the other hand, antenna miniaturization is usually achieved at the expense of efficiency, bandwidth, directivity and gain, but especially miniaturized antennas tends to present a practically omnidirectional radiation pattern. However, future regulations might require the use of adaptive directional antennas also for mobile terminals, due to the invaluable role of antennas in solving interference problems in multiuser wireless systems. The concept of antenna array superdirectivity^3^ can offer new perspectives for future wireless communication systems requiring spatial filtering, electromagnetic field exposure and energy consumption reduction, wireless systems cohabitation, and miniaturization.

Unlimited superdirectivity from arbitrarily small sources was first suggested to be attainable by Oseen^4^ in 1922, and later theoretically developed by Bouwkamp and De Bruijn^5^. For arrays with a certain number of elements, maximum possible directivity instead is limited, as shown by Uzkov^6^; in particular, a linear array of *N* uniformly spaced isotropic elements can have maximum endfire directivity approaching *N*^2^ for the inter-element distance decreasing to zero, in contrast to ordinary array theory^7^. However, to realise the maximum possible superdirectivity, the complex current at the input of each array element must be very tightly controlled. Limitations on superdirectivity of linear arrays of isotropic sources were deduced mathematically by introducing the array tolerance sensitivity and geometrical quality factor *Q*^8^. In practice, substantial coupling between individual radiators in an electrically small (size < *λ*) array will generate deviations of the current distribution from the required values and degrade the array performance. Alongside this, we find increases in reactive power (relative to the radiated power) and a rise in the quality factor (*Q*) of the array. Moreover, due to the small inter-element spacing and the large magnitudes of the excitation coefficients, ohmic losses increase and the antenna efficiency decreases very sharply. While these factors hampered the achievement of significant superdirectivity, interest in superdirective antennas and arrays resurfaced intermittently through the years (for details of these advances see, for example, reviews in^3,9^), and has continued up to recently^10–12^.

Recently, the first experimental demonstrations of superdirective arrays have been reported in the literature. In^13,14^ a tunable feed network has been included in the array to dynamically adjust the required currents of the individual radiating elements and alleviate the impact of the coupling. With a similar aim of controlling array element excitation, the use of metamaterial-based insulators and metamaterial phase shifting lines have been proposed in^15,16^, respectively. An alternative method for realising electrically small, superdirective arrays with much simpler feeding networks has been explored in^17,18^, where tunable components were no longer essential. The approach proposed in these works consists in forming a Yagi-like, parasitically coupled array antenna, where a single injection element is fed, whilst all the other elements are excited by their coupling to it. This method has been proved to be capable of achieving comparable directivity as the corresponding fully driven arrays^17^. Moreover, while the classic superdirective arrays used sub-resonant antennas closely spaced, this new class of superdirective arrays employs electrically small resonant elements, which allow for larger radiation resistance and thus larger efficiency than that of conventional superdirective arrays. A number of electrically small metamaterial antennas were subsequently proposed in order to realise miniaturised superdirective emitters exploiting the coupled array antenna concept^19–24^. In^20,22^ it was shown theoretically that far-field superdirectivity could be achieved using arrays formed by two or three split ring resonators (SRRs). These structures relied on the physics of slow electro- and magneto-inductive wave propagation^25,26^. These waves provide a high spatial frequency current distribution as is required for superdirectivity. More recently, a superdirective antenna design using coupled SRR dimers was reported^27^, which in both simulations and experimental results exhibited superdirective emission only a fraction of a decibel below the theoretical maximum for a two element dipole array. In order for a subwavelength device to achieve superdirectivity, it is required that the constituent resonators have high quality factors *Q* and strongly couple to each other with a high coupling constant $\kappa$. In fact it has been shown that conditions on excitation coefficients of superdirective arrays can be translated in the figure-of-merit $\kappa Q$ having to be proportional to the ratio of the wavelength *λ* and the unit cell size *d*^20,28^. However, this condition alone is sufficient to ensure a superdirective emission only for simple dipole arrays, but not in the case of coupled SRR dimers. For the SRRs there are two equivalent emitting dipoles, one magnetic from the circulating currents, and one electric from the charge polarisation at the split gap. The overall radiation properties of dimers formed by SRRs then depend strongly on how these radiation contributions combine in the far-field, which in turn is sensitive to the relative orientation of the SRRs.

The objective of this paper is to thoroughly investigate the conditions required for a dimer of coupled self-resonant SRRs, with only one driven element, to function as an electrically small superdirective antenna array. The orientation of the SRRs is assumed as a free parameter of the array and analytical, numerical, and experimental results are reported to shed light on the optimal design. In particular, identification of the SRR dimer configurations capable of superdirective emission is addressed by developing a complete analytical description of the radiation and directivity properties of two coupled SRRs. Our analytical model leverages an equivalent circuit representation of the coupled SRRs, which includes both their electric and magnetic coupling components, in combination with formulae espressing the far-field produced by a given distribution of currents. This model permits to rapidly estimate the directivity of an SRR dimer in an arbitrary configuration and thus to generate maps of directivity for any possible orientation of its constituent elements, as we anticipated in a short conference communication^29^. Findings deduced by means of our theoretical model are corroborated with full wave (FW) simulations and experiments. Insight gained in this work informed the design of a practical compact and highly efficient superdirective dimer of SRRs with an integrated feed structure, which has been manufactured by 3D printing fused filament deposition, as reported in^30^.

The principles of superdirective arrays and the physical requirements and constraints for an SRR dimer to provide superdirective radiation are discussed in the next section. Then, the analytical model to predict the directivity of a dimer of arbitrarily oriented SRRs is developed and maps of directivity are presented that allow identifying the dimer arrangement best suited to achieve superdirectivity. Simulated and measured results are provided to confirm the validity of predictions based on theory.

## Discussion

Whilst directivity in a phased array arises from the constructive interference of multiple sources signals, in superdirective emission one relies on destructive interference to cancel radiation in all but a small subset of the available directions. When aiming at superdiretivity in the endfire directions, the currents in a linear array of *N* dipoles must have a near-binomial distribution with the phases of adjacent elements being almost 180 degrees apart. This yields directivity proportional to *N*^2^ rather than just *N* as with a conventional phased array. For the case of a two-dipole array the maximum directivity of the phased-array is $D\simeq 3.5$, which is achieved at an inter-element spacing $d\simeq \lambda /3$, where *λ* denotes the free space wavelength; instead, the upper theoretical limit of directivity of a two dipole superdirective array is *D* = 5.25, which however is obtained for out-of-phase excitation of the dipoles in the limit of vanishing inter-element spacing (see Section S1 in the *Supplementary Information*). One of the main difficulties in achieving superdirectivity is indeed in the realization of such a current distribution varying fast on the scale of the free space wavelength.

**Figure 1:**
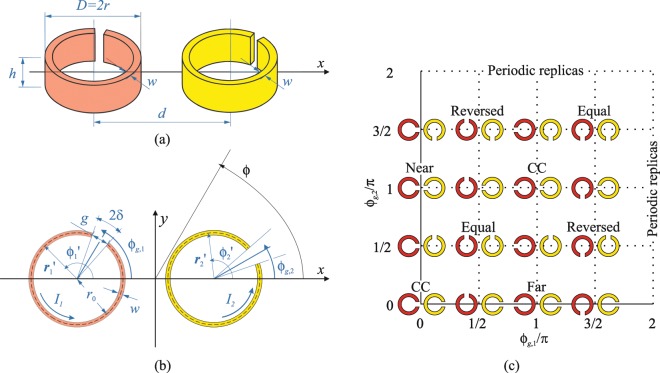
Dimer made of two coupled identical SRRs with arbitrary orientations: (**a**) perspective and (**b**) cross-sectional views. (**c**) Sample dimer configurations in the ${\phi }_{g,1}-{\phi }_{g,2}$ plane. The red shading indicates the ring which is assumed to be directly driven by an external source.

The meta-atom array candidate for superdirectivity, shown in Fig. 1(a,b), consists of two identical coplanar singly split ring resonators positioned along the *x* axis with their axes pointed along the *z* direction. They are separated by a distance *d* but each is rotated about the *z* axis at an arbitrary angle ${\phi }_{g,i}$, *i* = 1,2, with respect to the array axis. A few illustrative examples of dimer configurations corresponding to specific pairs of $({\phi }_{g,1},{\phi }_{g,2})$ values are displayed in Fig. 1(c). Instead of independently exciting each of the SRRs, the desired phase and amplitude relationship within the dimer is achieved by driving only one of the rings with an external source and relying on the coupling between the rings. In particular, the underlying mechanism is similar to the propagation of magneto-inductive (MI) waves, which are slow waves propagating on metamaterials comprising magnetically coupled elements such as SRRs^25,26^. Slow waves have very short wavelengths as compared to equal frequency light in free space so that excitation of slow waves makes the creation of a high spatial frequency current variation quite simple: they are provided by the standing slow waves in a finite structure. There is however an important difference with respect to standard MI waves in the quasi-static regime: since the meta-dimer is supposed to be formed with self-resonant SRR elements, currents along the rings are expected to be non-homogenous and significant charge densities will accumulate in the region of each gap. It is in fact well known that at the fundamental resonance SRRs with an odd number of gaps always exhibit overlapping in-plane electric and out-of-plane magnetic dipole responses^31,32^. As a consequence, the SRRs will be coupled through both their electric and magnetic fields. The coupling strength is generally quantified by means of a total coupling coefficient ${\kappa }_{T}$, whose definition is linked to the exchange of magnetic and electric energies between the resonators. As shown in^33,34^, the total coupling may be dominated by either its electric or magnetic part, depending on the relative orientation of the SRRs, and the overall coupling coefficient ${\kappa }_{T}$ is generally bound to be complex due non-negligible retardation effects which arise as a result of the sizes of the resonators and the distance between them being in the order of *λ*/6–*λ*/5.

**Figure 2:**
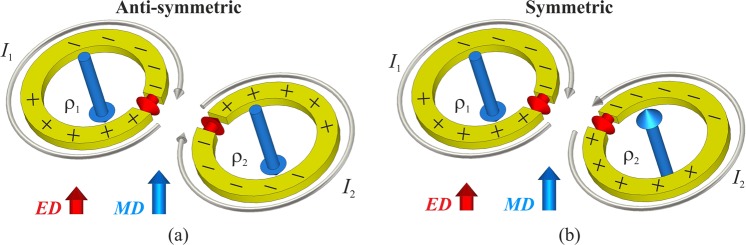
Schematic views of the surface charge distributions (denoted by ‘+’ and ‘−’ signs) along with the corresponding direction of circulation of the currents around the rings and the associated electric dipole (ED) and magnetic dipole (MD) at the resonances of a dimer in which the gaps of the constituent SRRs are facing each other. In this “near” configuration, overall coupling is maximum and is dominated by its electric part^31,34^. (**a**) Electrically anti-symmetric and (**b**) symmetric coupled resonance modes. The electric dipole moments are localized around the gap regions where charges accumulate, while the magnetic dipole moments approximately pass through the centres of the rings due to the circulating currents.

The coupling between two SRRs arranged in an arbitrary configuration has been studied with different approaches. The interaction energies have been determined, for example, using Lagrange’s equation of motion^35,36^ and in terms of the coupling of the in-plane electrical and the out-of-plane magnetic dipole moments associated with an SRR^31,37^. Circuit models have also been developed to qualitatively and quantitatively analyse SRRs and their coupling^34,38^. Moreover, based on a coupled mode formalism, general expressions for the coupling coefficient ${\kappa }_{T}$ valid for both conducting and dielectric resonators have been derived that make it possible to estimate the frequencies and fields of the coupled modes for arbitrarily oriented and spaced resonators^39–41^. These approaches enable the calculation of the characteristic parameters of a coupled system; in principle, they could be used to tailor the coupling and quality factor *Q* of the two SRR elements forming a meta-dimer to realize the array excitation prescribed for superdirectivity, given that these latter requirements can be translated into equivalent conditions on ${\kappa }_{T}$ and *Q*, as anticipated in^20,22^ and discussed in detail in^28^. However, if engineering the inter-element coupling is crucial to the design of a superdirective meta-dimer, this would actually suffice achieving superdirectivity only for dimers formed by simple dipole radiators. As recalled in the Introduction, in an SRR both electric and magnetic dipole resonance modes contribute to radiation, and since SRRs are usually not highly symmetric, the relative orientation of particles can also be of crucial importance. This effect is not completely addressed by existing theoretical models and can give rise to unexpected results. To clarify these aspects, we qualitatively examine the current and charge distributions in the sample dimer formed by two SRRs with the gaps facing each other, as shown in Fig. 2. In this configuration, which is referred to as “near”^33,34^, coupling is strong, predominantly electric and “real”, since the close proximity of the dominant charges minimises the effects of retardation^34^. The dimer, being a system of two coupled resonators, exhibits two resonances, whose frequencies are split as an effect of coupling. Figure 2(a) shows that at the electrically anti-symmetric resonance, the anti-symmetric distribution of charges correspond to surface currents in the two SRRs that circulate in the same direction (symmetric). At the electrically symmetric resonance of Fig. 2(b), distribution of charges is symmetric while surface currents in the SRRs flow in opposite senses (anti-symmetric). In other words, at both resonance frequencies, the two equivalent electric dipoles associated with the charges concentrated on the opposite sides of the gaps, will have opposite alignment, either parallel or anti-parallel, with respect to the pair of magnetic dipoles equivalent to the conduction currents in the SRRs. As a result, it appears that for a meta-dimer in this “near” configuration, in spite of the strong coupling that would facilitate the magnitude of the current of the parasitic element to be as large as that of the driven element, it would not be possible to simultaneously excite the pair of SRRs to have both electric and magnetic dipolar contributions to radiation approximately in anti-phase, as required for superdirectivity. Similar reasoning and conclusions could be applied to the dimer configuration referred to as “far” (see Fig. 1(c)), where the coupling between the rings is dominated by the magnetic part. However, the near field interaction of two SRRs is generally controlled by both their electric and magnetic coupling, which can oppose or reinforce each other depending on the relative orientation of the SRRs. In this context, it would be difficult to solely rely on physical intuition for the selection of the dimer configuration most effective to achieve superdirectivity; therefore we aimed at developing an analytical model for the rapid and sufficiently accurate assessment of the radiation properties of dimers of coupled SRRs with arbitrary orientations, which is described in the next section.

## Methods

### Analytical model of a dimer of arbitrarily oriented SRRs

**Figure 3:**
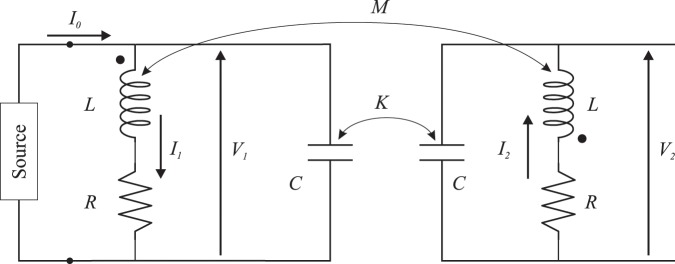
Equivalent circuit of the meta-dimer made of two identical arbitrarily orientated SRRs shown in Fig. 1. Both electric (*K*) and magnetic (*M*) couplings are assumed.

Our analytical model is based on combining the equivalent circuit description of the coupled resonators with closed-form expressions for the surface charge and current densities along a circular SRR and with the standard formulae for radiation of currents. Our coupled SRRs are analysed using their equivalent *LCR* circuit in Fig. 3. This is a modification of the circuit model introduced in^34^ for the type of feed adopted in this analysis, which consists of an ideal, either current or voltage, source connected across the split gap of one of the two SRRs. The electric and magnetic coupling between the resonators are modelled by introducing the mutual capacitance (*K*) and inductance (*M*), which account for the effect of retardation (see Section S2 of the *Supplementary Information*). The currents *I*_1_ and *I*_2_ in Fig. 3 represent the total currents circulating in the SRRs, including their displacement currents, which are independent of space, and coincide with the excitation coefficients of the array. Retardation is neglected at the level of the single resonator. *I*_0_ is the current provided by the source (if the source is a current generator *I*_0_ will be constant, while it will change according to the input impedance of the SRR dimer if a voltage source is considered; however, this is irrelevant for our analysis, where only the ratio of the excitation coefficients is of interest).

Assuming a harmonic time variation of the type exp(−*iωt*), the equivalent circuit in Fig. 3 can be solved for *I*_1_ and *I*_2_ as

1 $$\frac{{I}_{1}}{{I}_{0}}=\frac{{\omega }^{2}KM-i\omega C(R-i\omega L)+1}{F(\omega )},\,\frac{{I}_{2}}{{I}_{0}}=\frac{{\omega }^{2}MC-i\omega K(R-i\omega L)}{F(\omega )}$$

where

2 $$F(\omega )={\omega }^{3}({K}^{2}-{C}^{2})[({M}^{2}-{L}^{2})\omega -2iRL]+{\omega }^{2}[{R}^{2}({K}^{2}-{C}^{2})-2(CL+KM)]-2i\omega RC+1$$

The derivation of these expressions is detailed in Section S2 of the *Supplementary Information*. Introducing the quantities

3 $${\omega }_{0}=\frac{1}{\sqrt{LC}},\,{Q}_{0}=\frac{{\omega }_{0}L}{R},\,Q=\frac{\omega L}{R}=\frac{\omega }{{\omega }_{0}}{Q}_{0},$$

and the electric and magnetic coupling coefficients

4 $${\kappa }_{E}=\frac{K}{C},\,{\kappa }_{H}=\frac{M}{L},$$

the ratio of the total currents *I*_2_/*I*_1_ that can be derived from (1) can be cast in the form

5 $$\frac{{I}_{2}}{{I}_{1}}=\frac{\frac{{\omega }^{2}}{{\omega }_{0}^{2}}[{\kappa }_{H}-{\kappa }_{E}(1+\frac{i}{Q})]}{1+\frac{{\omega }^{2}}{{\omega }_{0}^{2}}{\kappa }_{E}{\kappa }_{H}-\frac{{\omega }^{2}}{{\omega }_{0}^{2}}(1+\frac{i}{Q})}$$

Considering that $|{\kappa }_{E}|,|{\kappa }_{H}|\le 1$, such that it is reasonable to assume $|{\kappa }_{E}{\kappa }_{H}|\ll 1$, and that $Q\gg 1$, (5) can be simplified as

6 $$\frac{{I}_{2}}{{I}_{1}}\simeq -\frac{{\kappa }_{T}}{1-\frac{{\omega }_{0}^{2}}{{\omega }^{2}}+i\frac{{\omega }_{0}}{\omega }\frac{1}{{Q}_{0}}}$$

where ${\kappa }_{T}={\kappa }_{H}-{\kappa }_{E}$ in accordance with the formula for the total coupling coefficient derived using Lagrange’s equation of motion^36^ and coupled mode theory^39,41^.

To achieve superdirectivity, the ratio of the total currents in the SRRs *I*_2_/*I*_1_ needs to be the same as the ratio of the excitation coefficients required for an array of two dipoles separated by the same distance *d*. In particular, for an array of two dipoles aligned along the *x* axis and with their dipole moments in the *z* direction, for maximization of the directivity $D({\theta }_{0},{\phi }_{0})$ in the two endfire directions $({\theta }_{0}={90}^{^\circ })$ the optimal excitation coefficients of a conventional superdirective dipole array ${I}_{j}^{{\rm{opt}}}$, *j* = 1,2, can be found to be^14^

7 $${I}_{1}^{{\rm{opt}}}=\frac{1-\frac{3}{2}p{e}^{\mp ikd}}{(\frac{2}{3}-\frac{3}{2}{p}^{2})},\,{I}_{2}^{{\rm{opt}}}=\frac{1}{p}(1-\frac{2}{3}{I}_{1}^{{\rm{opt}}})$$

where

8 $$p=\frac{\sin (kd)}{kd}[1-\frac{1}{{(kd)}^{2}}]+\frac{\cos (kd)}{{(kd)}^{2}}$$

As shown in^28^, by equating (6) to the ratio ${a}_{02}/{a}_{01}$, an insightful relation can be established between requirements for superdirectivity and the coupling and quality factor of the constituent elements of a meta-dimer. In fact, from the expansion in Taylor series of the ratio of the excitation coefficients for small *kd* we have

9 $$\frac{{I}_{1}^{{\rm{opt}}}}{{I}_{2}^{{\rm{opt}}}}=\frac{1-\frac{3}{2}p{e}^{\pm ikd}}{{e}^{\pm ikd}-\frac{3}{2}p}=-\,1\mp i\frac{2}{5}kd+\frac{2}{25}{(kd)}^{2}\pm i\frac{323}{10500}{(kd)}^{3}+O({(kd)}^{4})$$

By comparing (6) with the first two terms of (9), we deduce that for two SRRs to behave as a superdirective array requires

10 $$(-\frac{{I}_{1}}{{I}_{2}})=\frac{1}{{\kappa }_{T}}(1-\frac{{\omega }_{0}^{2}}{{\omega }^{2}}+i\frac{{\omega }_{0}}{\omega }\frac{1}{{Q}_{0}})\simeq 1\pm i\frac{2}{5}kd$$

Since due to retardation $${\kappa }_{T}$$ can be in general a complex quantity, we can write $${\kappa }_{T}=\Re ({\kappa }_{T})+i\Im ({\kappa }_{T})=$$$$|{\kappa }_{T}|\exp (i{\phi }_{{\kappa }_{T}})$$, and by equating the real and imaginary parts of (10), we deduce the following conditions for a real solution *ω* of (10) to exist

11 $$\{\begin{array}{l}1-\frac{{\omega }_{0}^{2}}{{\omega }^{2}}=-[\Re ({\kappa }_{T})\mp \Im ({\kappa }_{T})\frac{2}{5}kd]\\ \frac{{\omega }_{0}}{\omega }\frac{1}{{Q}_{0}}=[\Im ({\kappa }_{T})\pm \frac{2}{5}kd\Re ({\kappa }_{T})]\end{array}$$

As far as *kd* is small and it can be assumed that ${Q}_{0}\gg 1$ and $|{\kappa }_{T}|\le 1$, using the approximation $\sqrt{1-x}\simeq 1-x/2$ for $|x|\ll 1$ in the first condition in (11), we obtain

12 $$\{\begin{array}{l}{\omega }_{{D}_{{\rm{\max }}}}\simeq \frac{{\omega }_{0}}{1-\frac{1}{2}|{\kappa }_{T}|[\cos \,{\phi }_{{\kappa }_{T}}\mp \frac{2}{5}kd\,\sin \,{\phi }_{{\kappa }_{T}}]}\\ \frac{1}{|{\kappa }_{T}|\cos \,{\phi }_{{\kappa }_{T}}{Q}_{0}}-\,\tan \,{\phi }_{{\kappa }_{T}}\simeq \pm \frac{2}{5}kd\end{array}$$

**Figure 4:**
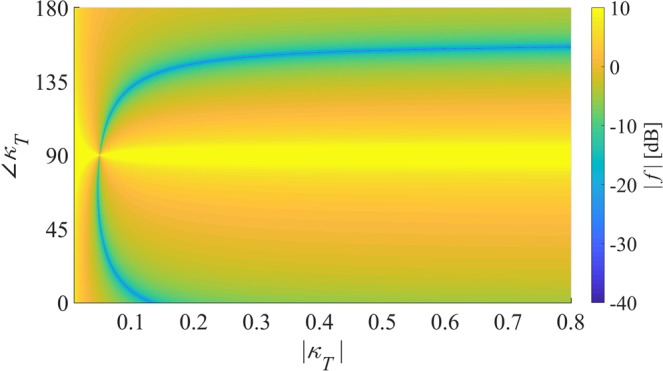
Map of the residual value of the second of the conditions (12) $f({\kappa }_{T})={(|{\kappa }_{T}|\cos {\phi }_{{\kappa }_{T}}{Q}_{0})}^{-1}-\tan {\phi }_{{\kappa }_{T}}-2kd/5$, in logarithmic scale, for varying magnitude and phase of ${\kappa }_{T}$ when the distance between the radiating elements is set to $d=0.15\lambda$ and ${Q}_{0}=20$.

The residual value of the second of the conditions (12) is shown in dB scale in Fig. 4 against the magnitude and phase of the total coupling coefficient ${\kappa }_{T}$ when *d* = 0.15*λ* and *Q*_0_ = 20. All regions of the map where the residual value is smaller than −10 dB correspond to coupling conditions substantially suitable for superdirectivity. At ${\phi }_{{\kappa }_{T}}\simeq {90}^{^\circ }$, coupling is lossy, as it could occur in an absorbing medium, and, not surprisingly, this condition is the most unfavourable for superdirectivity. From this map it can be further observed that requirements on the strength of ${\kappa }_{T}$ can be relaxed in comparison to the case when ${\kappa }_{T}$ is real (e.g., for “near” and “far” dimer configurations) provided that its phase can be suitably engineered by adjusting the relative position and orientations of the elements of the dimer. The equivalent circuit developed in this work enables the rapid assessment of the ratio of the currents in the SRR dimer and its congruency with the corresponding reference value for an ideal superdirective array of two dipoles (i.e. condition 12). However, the latter condition is necessary but not sufficient for superdirectivity, as discussed in the previous section and further clarified in the second part of this work. The numerical solution of the dimer equivalent circuit and the calculation of the SRR currents require the preliminary estimation of the parameters *R*,*L*,*C*, of a single SRR and of the coupling terms *M*,*K* characterizing the various dimer configurations; these steps are described in detail in Section S2 of the *Supplementary Information*.

### Directivity of SRR meta-dimers

Now, the estimation of the SRR currents provided by the equivalent circuit presented in the preceding section is combined with radiation formulae, so as to form a completely analytical tool capable of rapidly predicting the directivity of a dimer formed by arbitrarily oriented elements. Once the total currents *I*_1_ and *I*_2_, are known, the far-field radiated by the dimer can be calculated in standard spherical coordinates $(r,\theta ,\phi )$ as^42^

13 $$[\begin{array}{c}{E}_{\theta }(r,\theta ,\phi )\\ {E}_{\phi }(r,\theta ,\phi )\end{array}]=i\frac{\omega \mu {r}_{0}{e}^{ikr}}{4\pi r}{I}_{1}[\begin{array}{c}{E}_{\theta }^{0}(\theta ,\phi )\\ {E}_{\phi }^{0}(\theta ,\phi )\end{array}]$$

where

14 $$[\begin{array}{c}{E}_{\theta }^{0}(\theta ,\phi )\\ {E}_{\phi }^{0}(\theta ,\phi )\end{array}]=[{e}^{i(\frac{kd}{2}\sin \theta \cos \phi )}{S}_{1}(\theta ,\phi ;{\phi }_{g,1})+\frac{{I}_{2}}{{I}_{1}}{e}^{-i(\frac{kd}{2}\sin \theta \cos \phi )}{S}_{2}(\theta ,\phi ;{\phi }_{g,2})]$$

and

15 $${S}_{j}(\theta ,\phi ;{\phi }_{g,j})=[\begin{array}{c}{S}_{j}^{\theta }\\ {S}_{j}^{\phi }\end{array}]={\int }_{\delta +{\phi }_{g,j}}^{2\pi -\delta +{\phi }_{g,j}}{I}_{n}(\phi ^{\prime} -{\phi }_{g,j})[\begin{array}{c}\cos \,\theta \,\sin (\phi -\phi ^{\prime} )\\ cos(\phi -\phi ^{\prime} )\end{array}]{e}^{-ik{r}_{0}\sin \theta \cos (\phi -\phi ^{\prime} )}d\phi ^{\prime} ,\,j=1,2$$

In (15) ${I}_{n}(\phi )$ is the normalized current tapering function of an SRR which as in^34^, based on the expressions of the electric field of an infinitely long conducting circular cylinder with an infinitesimal gap derived in^43^ and then extended to a ring of finite size^44^, can be written as

16 $${I}_{j,n}(\phi ;{\phi }_{g,j})={I}_{n}(\phi ;{\phi }_{g,j}){\hat{\phi }}_{j}=\{1-\frac{\mathrm{ln}[\sin (\frac{\phi -{\phi }_{g,j}}{2})]}{\mathrm{ln}[\sin (\frac{\delta }{2})]}\}{\hat{\phi }}_{j},\,j=1,2$$

where ${\hat{\phi }}_{j}$ are local azimuthal unit vectors; the SRR currents flow in the angular regions $\delta +{\phi }_{g,j}\le \phi \le 2\pi -\delta +{\phi }_{g,j}$ with $2\delta =2\arcsin [g/(2{r}_{0})]$, *j* = 1,2, corresponding to the angular arc of the gap *g*, and *r*_0_ = *r*−*w*/2 being the radius of the filament current localized at the centre of the SRR cross section (cf. Fig. 1). From (14) and (15) the radiation intensity *U* can be calculated as according to the usual definition^42^

17 $$U(\theta ,\phi )=\frac{{r}^{2}}{2\zeta }[{|{E}_{\theta }(r,\theta ,\phi )|}^{2}+{|{E}_{\phi }(r,\theta ,\phi )|}^{2}]=\frac{\omega \mu {r}_{0}{I}_{1}}{4\pi }\frac{1}{2\zeta }[{|{E}_{\theta }^{0}(\theta ,\phi )|}^{2}+{|{E}_{\phi }^{0}(\theta ,\phi )|}^{2}]$$

where $\zeta$ is the intrinsic impedance of the medium; the directivity in a given direction $({\theta }_{0},{\phi }_{0})$ can then be written as

18 $$D({\theta }_{0},{\phi }_{0})=\frac{4\pi U({\theta }_{0},{\phi }_{0})}{{P}_{rad}}=\frac{4\pi [{|{E}_{\theta }^{0}({\theta }_{0},{\phi }_{0})|}^{2}+{|{E}_{\phi }^{0}({\theta }_{0},{\phi }_{0})|}^{2}]}{{\int }_{0}^{2\pi }{\int }_{0}^{\pi }[{|{E}_{\theta }^{0}(\theta ,\phi )|}^{2}+{|{E}_{\phi }^{0}(\theta ,\phi )|}^{2}]\sin \,\theta d\theta d\phi }$$

On examining (18) together with (14) and (15), it can be noted that, as expected, calculation of directivity only requires the knowledge of the ratio of the total currents (i.e., of the excitation coefficients), which can be estimated through the equivalent circuit model, and the current tapering function.

## Results

### Mapping the directivity of SRR meta-dimers

The analytical model we have developed allows the rapid calculation of the directivity of our dimer, regardless of the orientation of its two SRRs, and can be used to create maps of the maximum possible directivity for all dimer configurations generated as the SRR orientations are varied. In our calculations the two identical SRRs have the following dimensions: *D* = 22 mm, *g* = 2 mm, *w* = 0.8 mm, *h* = 5 mm, and *d* = 27 mm. The resonance of this SRR in isolation is predicted to occur at ~1.96 GHz by simulation with CST Microwave Studio (MWS)^45^. The SRRs are positioned along the *x* axis with their centres at (±*d*/2,0,0). Only the SRR at (−*d*/2,0,0) is fed with an ideal voltage source connected at its gap, whose location is specified by the angle ${\phi }_{g,1}$ (cf. Fig. 1).

**Figure 5:**
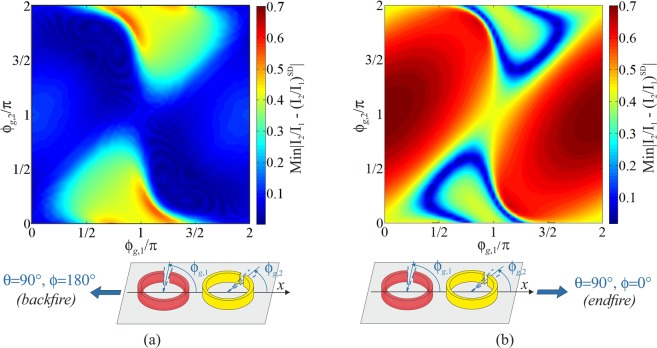
Maps of the minimum of the magnitude of the difference between the ratio of the total currents in an SRR dimer and the ratio of the ideal excitation coefficients for a two dipole superdirective array (7) over the frequency range 1.7–2.1 GHz vs the angular orientations of the dimer constituent elements for maximum radiation in the backfire and endfire directions sketched at the bottom of the maps: (**a**) $\theta ={90}^{^\circ },\phi ={180}^{^\circ }$; (**b**) $\theta ={90}^{^\circ },\phi ={0}^{^\circ }$. The SRRs have dimensions *D* = 22 mm, *g* = 2 mm, *h* = 5 mm, *w* = 0.8 mm.

Before proceeding with studying the directivity of the dimer, it is interesting to preliminary assess how effective is the coupling mechanism between the SRRs due to slow magneto- and electro-inductive waves in producing the fast varying current distribution needed for superdirectivity. To this aim, Eq. (1) is used to calculate the ratio of the total currents in the SRRs for all possible orientations of the SRRs over a wide frequency range around the resonance of an isolated SRR element, specifically, the interval 1.7–2.1 GHz. These values are compared with the ratio of the ideal excitation coefficients (7) required for an array of two dipoles to exhibit superdirective radiation, in particular, in the backward and forward endfire directions $\theta ={90}^{^\circ },\phi ={180}^{^\circ }$ and $\theta ={90}^{^\circ },\phi ={0}^{^\circ }$, which in the following will be simply referred to as “backfire” and “endfire”, as sketched in Fig. 5. The minimum, over the considered frequency range, of the magnitude of the difference of these complex quantities is depicted in the maps of Fig. 5 against the angles ${\phi }_{g,1}$ and ${\phi }_{g,2}$ specifying the SRR orientations. The maps in Fig. 5(a,b) are almost complementary and predict that a large variety of SRR dimer configurations can accomplish, at some frequency in the neighbourhood of the resonance, the necessary phase relationship between the excitation coefficients to achieve superdirectivity in the backfire direction $\theta ={90}^{^\circ },\phi ={180}^{^\circ }$, while only a small subset of dimer arrangements can be properly excited to realize superdirectivity in the endfire direction $\theta ={90}^{^\circ },\phi ={0}^{^\circ }$. However, as pointed out in the Discussion section, implementing the prescribed excitation is a necessary, but not sufficient condition for superdirectivity when the dimer constituent elements are SRRs rather than simple dipoles, and thus maps in Fig. 5 only provide limited guidance for the design.

**Figure 6:**
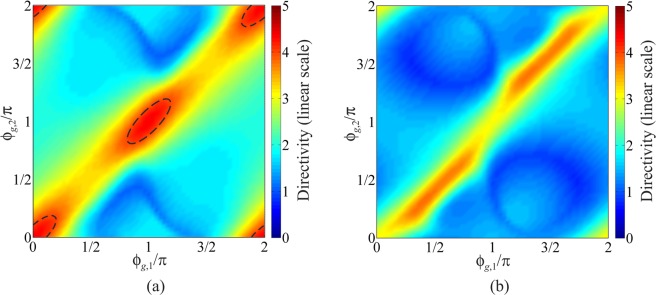
Maps of maximum directivity of an SRR dimer vs the angular orientations of its constituent elements for backfire and endfire maximum radiation (see Fig. 5): (**a**) $\theta ={90}^{^\circ },\phi ={180}^{^\circ }$; (**b**) $\theta ={90}^{^\circ },\phi ={0}^{^\circ }$. SRR dimensions are: *D* = 22 mm, *g* = 2 mm, *d* = 5 mm, *w* = 0.8 mm, *h* = 5 mm. The black dashed contour lines visible in the first map correspond to *D* = 4.

Next, the directivity of the SRR dimer is studied by combining Eqs. (14), (15) and (18), together with (1) to compute the maps shown in Fig. 6. These results thus take into account both the tapering of the current density on the SRRs, and accordingly the dual electric and magnetic dipole mode contribution to radiation in action in each ring, and the specific ratio of currents arising in the SRRs as a result of their coupling, which is configuration dependent. In particular, the maps in Fig. 6 were created by finding the maximum directivity achieveable by the dimer in the backfire and endfire directions from 1.7 to 2.1 GHz for each combination of SRR orientations ${\phi }_{g,1}$ and ${\phi }_{g,2}$ $(0\le {\phi }_{g1},{\phi }_{g2}\le 2\pi )$. The space of dimer configurations is sampled varying the angles in steps of $\Delta {\phi }_{g1}=\Delta {\phi }_{g2}={6}^{^\circ }$ for each element, which corresponds to a total of 61 × 61 = 3721 different arrays analysed.

The map in Fig. 6(a) for backfire radiation, over which the contour lines corresponding to *D* = 4 are superimposed, shows a few local directivity maxima for ${\phi }_{g,1}={\phi }_{g,2}=n\pi$, *n* = 0,1 (along with their periodic repetitions). These correspond to two, very similar, dimer configurations where the split gaps are both in the same direction along the array axis, as in two letters “C” side by side for ${\phi }_{g,1}={\phi }_{g,2}=0$, or their mirror images for ${\phi }_{g,1}={\phi }_{g,2}=\pi$; these latter are denoted henceforth as the “CC” configuration. The directivity maximum for these “CC” configurations predicted by the analytcal model is *D*_max_ = 4.34, which is quite close to the theoretical maximum for an equivalent dipole array with the same spacing $(kd\simeq 1)$ of 4.9. We also see enhanced directivity in the opposite endfire direction $\theta ={90}^{^\circ },\phi ={0}^{^\circ }$, in Fig. 6(b), along which however the largest directivity is only ${D}_{{\rm{\max }}}\simeq 3.8$. Since the driven resonator is always located at (−*d*/2,0,0), optimum superdirectivity is always obtained in a configuration where the parasitic element acts as a reflector^18^. Whilst we have focused on investigating the effect of SRR orientations on the dimer emission, assuming for simplicity identical elements, selecting different SRR radii along with different widths of the gaps would provide additional degree of freedom facilitating two equally important objectives: i) fine tuning of the magnetic and electric coupling to achieve a closer fulfillment of conditions for superdirectivity, and thus values of directivity tending to reach the theoretical maximum; ii) impedance matching to a practical feed structure. While addressing these points is beyond the scope of this work, the applicability of the model for non-identical SRRs is illustrated through the examples presented in Section S3 of the *Supplementary Information*.

Furthermore, it is also noteworthy that mapping the dimer directivity by approximating each SRR as a uniform filament current loop, which in the far-field is simply equivalent to a magnetic dipole, would not provide any additional information about the actual radiation performance of the dimer with respect to the maps in Fig. 5, because of the complete circular symmetry of the element pattern in the plane of the rings. In fact, under this simplifying assumption for the SRRs, maps of directivity would appear to be just complementary to maps in Fig. 5, i.e. the closer the excitation coefficients are to the theoretical values for superdictivity (blue-shaded regions in Fig. 5), the higher the directivity of the array would be estimated, as shown in Section S3 of the *Supplementary Information*.

The above results of the analytical model, taking advantage of incorporating a realistic tapering of the current density on the resonant SRRs both to predict the inter-element coupling and their combined radiation properties, are consistent with FW simulations and experiments. These results all confirm that only the “CC” configuration for dimers made of coupled SRRs leads to superdirectivity, as previously briefly reported in^27^ and illustrated in detail in the next section. It is noteworthy that from a physical standpoint this finding appears justified based on the observation that the “CC” meta-dimer is the only one providing simultaneous, approximately in anti-phase, excitation of both the in-plane electric and out-of-plane magnetic dipole responses of each of the SRRs forming the dimer, in full consistency with superdirectivity requirements.

### Numerical and experimental validation

FW simulations with CST MWS and experiments have been carried out to validate the results of the analytical model and assess the radiation performance of coupled SRR dimers at variable separation between the elements and for elements of different sizes. Results providing evidence of the superdirective properties of the “CC” arrangement are reported in the following.

**Figure 7:**
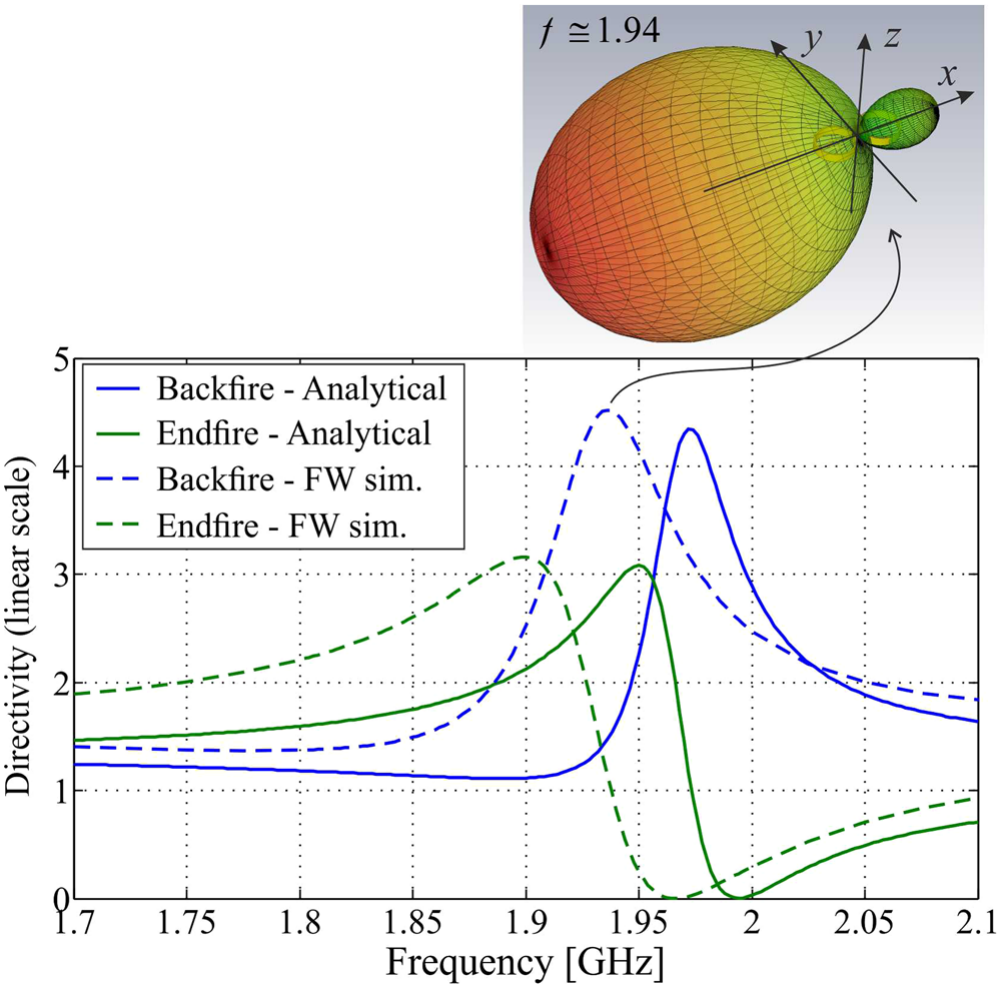
Directivity against frequency as predicted by the proposed analytical model in the backfire and endfire directions $\theta ={90}^{^\circ },\phi ={180}^{^\circ }$ (blue solid line) and $\theta ={90}^{^\circ },\phi ={0}^{^\circ }$ (green solid line) compared with corresponding FW simulation results (blue and green dashed lines) for a “CC” dimer formed by two identical SRRs with dimensions: *D* = 2*r* = 22 mm, *g* = 2 mm, *h* = 5 m, *w* = 0.8 mm, and *d* = 27 mm. In the top inset is shown the simulated directivity pattern of the dimer at the frequency of maximum directivity $f\simeq 1.94\,{\rm{GHz}}$.

Firstly, the directivity obtained analytically is compared with results of FW simulations. In the simulations, as well as in the experiments, the “CC” dimer is fed by a small magnetic loop probe placed vertically at the centre point of one of the SRR elements. The constituent SRR elements have dimensions *D* = 22 mm, g = 2 mm, *g* = 5 mm, *w* = 0.8 mm, and *d* = 27 mm. Figure 7 shows the directivity as a function of frequency in the backfire and endfire directions, $\theta ={90}^{^\circ },\phi ={180}^{^\circ }$ and $\theta ={90}^{^\circ },\phi ={0}^{^\circ }$, respectively. As apparent, the analytical model is consistent with simulations and reproduces very well the trend of directivity in both considered directions of radiation, there being only a <2% shift of frequency between the analytical and simulated peaks of directivity. Moreover, the maximum value of backfire directivity achieved by the dimer according to FW simulations is *D*_max_ = 4.52 $(f\simeq 1.94\,{\rm{GHz}})$, which is very close to the analytical estimation, *D*_max_ = 4.34 $(f\simeq 1.97\,{\rm{GHz}})$. The 3D directivity pattern of the dimer simulated at the frequency of maximum directivity, shown in the inset on top of Fig. 7, confirms the unidirectional pencil beam radiation of the dimer, with a substantially symmetric angular distribution of the radiated power around the backfire direction.

**Figure 8:**
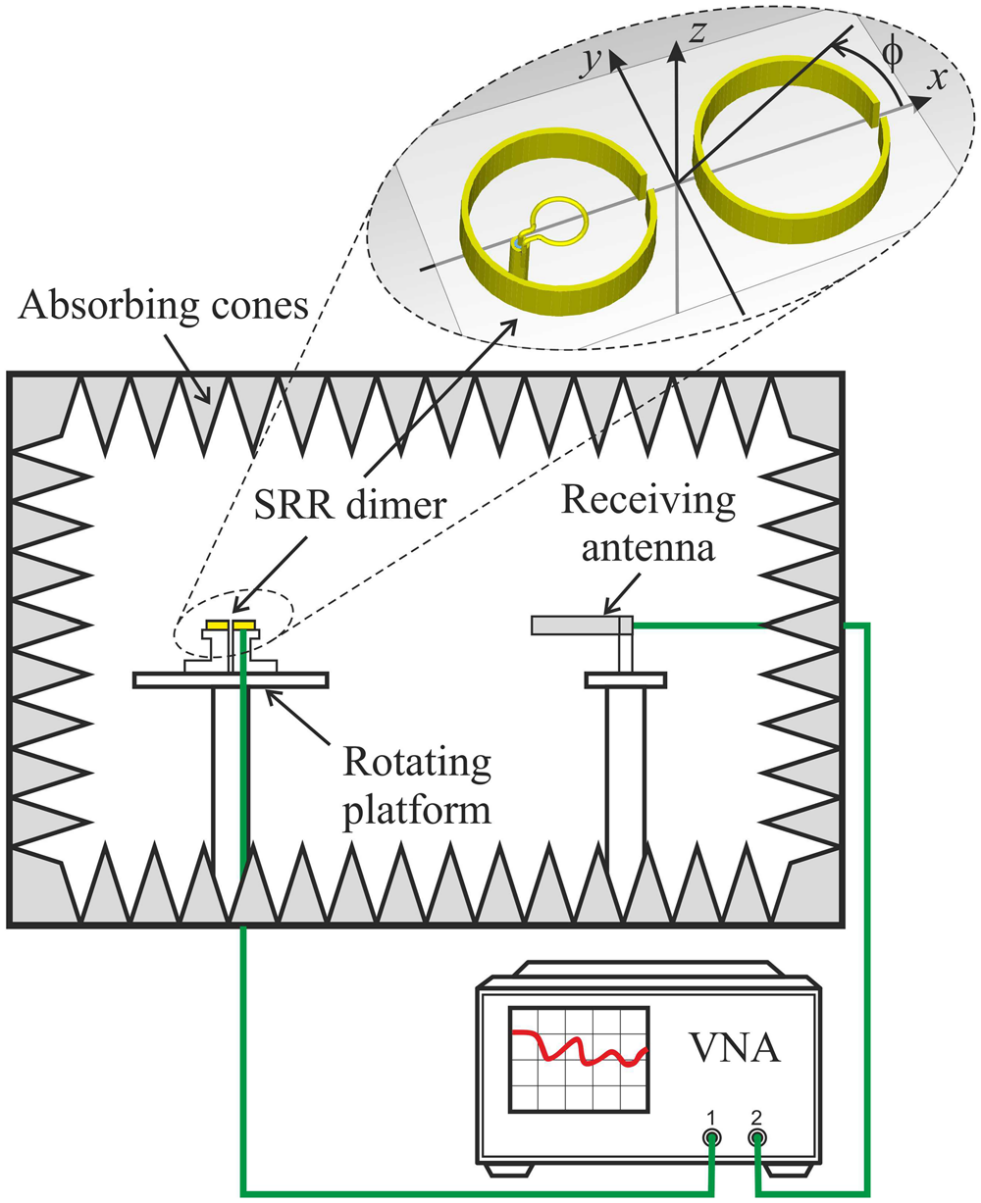
Transmission measurement setup in an anechoic chamber. The SRR dimer with “CC” disposition of the radiating elements is shown in the top inset.

Next, the experimental data are presented. The measurement setup is shown in Fig. 8. The measurements have been performed in an anechoic chamber where the radiated field spectrum has been evaluated over a complete sweep of the azimuthal angle. The SRRs forming the dimer were glued onto separate slabs of balsa wood placed in turn on top of two ABS stands, one of which was attached to a precision positioning stage providing adjustable inter-element spacing. The test fixture was mounted on a motorized rotating platform with its centre aligned with the rotating axis of the platform. For convenience of operation and improved visualization of measured data, the field was recorded starting from $\phi ={90}^{^\circ }$. The dimer with its small magnetic loop feed functioned as the transmitting antenna. A vector network analyser (VNA) drove the feeding magnetic loop probe and the receiving antenna (logperiodic), positioned at large enough distance from the dimer to ensure far-field conditions. The VNA measured the transmission coefficient *s*_21_ between the two antennas for each frequency and angle under computer control. The background transmission in the absence of the SRR dimer was always measured first and then subtracted from the measurement of the field transmitted by the complete antenna.

**Figure 9:**
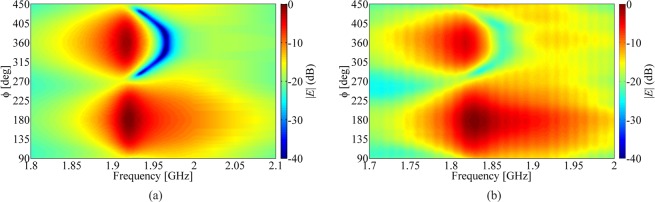
Maps of the magnitude of far-field radiation of the coupled SRR dimer in the “CC” configuration against frequency and the angle of observation *ϕ* in the plane of the dimer (cf. Fig. 8): (**a**) FW simulation; (**b**) measurements. The SRRs have dimensions *D* = 22 mm, *g* = 2 mm, *h* = 5 mm, *w* = 0.8 mm, and *d* = 27 mm.

**Figure 10:**
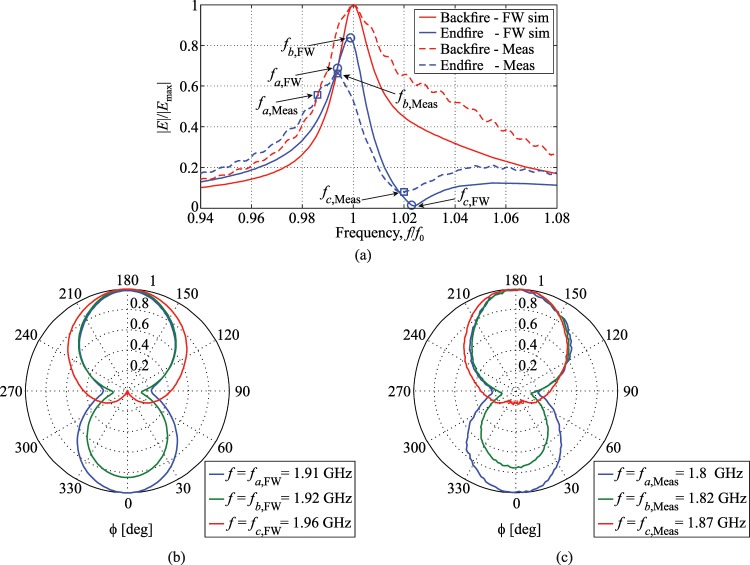
(**a**) Magnitude against frequency of the simulated and measured electric field, normalized to their respective maximum values over all observed angles, radiated in the backfire $(\phi ={180}^{^\circ })$ and endfire $(\phi ={0}^{^\circ })$ directions by a dimer of SRRs in the “CC” configuration with dimensions: *D* = 22 mm, *g* = 2 mm, *h* = 5 mm, *w* = 0.8 mm, and *d* = 27 mm (cf. Fig. 1 and inset in Fig. 8). (**b**) Simulated and (**c**) measured radiation patterns at the different frequencies (19) marked in graph (**a**). The downward frequency shift of the measured field is seemingly due to the influence of the wooden slab $({\varepsilon }_{r}\simeq 1.3)$ on top of which the SRRs are placed in the test fixture.

The map of the far-field measured from the dimer, normalized to its maximum value, is shown in Fig. 9 as a function of frequency and of the transmission/observation angle *ϕ*, in comparison with the corresponding FW simulation results by CST MWS. It is noted that measured and simulated field values are in reasonable agreement and show very similar frequency and angular dependencies, apart from a downward shift of frequency in the measured transmitted field, possibly due to the influence of the test fixture, in particular, the wooden slab on top of which the SRRs are placed, whose permittivity is estimated to be ${\varepsilon }_{r}\simeq 1.3$. Both measurements and simulations desmonstrate that there are two peaks in the far-field map, which occur at frequencies that are slightly shifted downwards and upwards with respect to the antisymmetric resonance frequency of the coupled SRRs. One peak corresponds to the endfire direction where the parasitic SRR acts as a director $(\phi ={360}^{^\circ }/{0}^{^\circ })$, and the other to the backfire direction for which the parasitic SRR element acts as a reflector $(\phi ={180}^{^\circ })$. The comparison between measurements and simulations of the dimer radiation is presented in more detail in Fig. 10. In Fig. 10(a) the magnitude of the measured and simulated transmitted field, normalized with respect to their respective maximum values (at $\phi ={180}^{^\circ }$), in the directions $\phi ={180}^{^\circ }$ and $\phi ={0}^{^\circ }$ are plotted against the normalized frequency *f*_*n*_ = *f*/*f*_0_, where *f*_0_ denotes the frequency at which the peak of $|E(f;\phi ={180}^{^\circ })|$ occurs; this comparison further highlights the similar trends of simulated and measured data. A few points are marked on the curves of $|E(f;\phi ={180}^{\circ })|$ and $|E(f;\phi ={360}^{\circ })|$ in Fig. 10(a), corresponding to the frequency points identified by the conditions

19 $$\begin{array}{c}{f}_{a,x}:|E({f}_{a,x};\phi ={0}^{^\circ })|=\,{\rm{\max }}|E(f;\phi ={0}^{^\circ })|\\ {f}_{b,x}:|E({f}_{b,x};\phi ={180}^{^\circ })|-|E({f}_{b,x};\phi ={0}^{^\circ })|=\,{\rm{\min }}(|E(f;\phi ={180}^{^\circ })|-|E(f;\phi ={0}^{^\circ })|)\\ {f}_{c,x}:|E({f}_{c,x};\phi ={180}^{^\circ })|-|E({f}_{c,x};\phi ={0}^{^\circ })|=\,{\rm{\max }}(|E(f;\phi ={180}^{^\circ })|-|E(f;\phi ={0}^{^\circ })|)\end{array}$$

with *x* = FW, Meas referring to simulated and measured data, respectively. Figure 10(b,c) show the numerical and experimental radiation patterns at these sample frequencies, which closely resemble each other, thus corroborating the validity of the obtained results. Upon the assumption that the radiated beam of the SRR dimer be rotationally symmetrical in a plane orthogonal to its pointing direction, which is fully supported by numerical simulations, at least in the neighbourhood of the frequency of maximum directivity (see inset in Fig. 7), the experimental directivity can be estimated with

20 $$D(f;{\theta }_{0})=\frac{2{|E(f;{\theta }_{0})|}^{2}}{{\int }_{0}^{\pi }\,{|E(f;\theta )|}^{2}\,\sin \,\theta d\theta }$$

By applying (20) with the available measured pattern data, the dimer has been found to exhibit its maximum directivity in the backfire direction $\phi ={180}^{^\circ }$ at a frequency slightly above the antisymmetric resonance of the coupled SRRs, $f\simeq 1.85\,{\rm{GHz}}$. Here it reaches ${D}_{m}\simeq 4.65$, which is in line with our FW results and very close to the theoretical maximum *D*_*m*_ = 4.9 for the corresponding dipole array with the same distance between the elements. This outcome is also consistent with theoretical results and previous finding that optimum superdirectivity of a two-element parasitically coupled array is obtained in the configuration where the parasitic element acts as a reflector^18^, providing a stronger base for its general validity. The proposed superdirective array is effectively scalable to any frequency/higher frequencies. In particular, extension of the “CC” meta-dimer design up to hundreds of GHz is perfectly feasible by simply adopting SRRs with a bulkier cross section in order to reduce both ohmic losses and the additional kinetic inductance accounting for the growing inertia of the current conducting electrons in metals at higher frequencies; this simple expedient helps enhancing the magnetic response of the miniaturized SRRs and preserving the strength of magnetic coupling between elements at high frequencies, which is essential for the selected parasitically coupled array design^46^.

### Concluding remarks

The potential for a dimer of coupled self-resonant SRRs to function as an electrically small superdirective antenna array has been investigated analytically, by simulations, and experiments. To support physical understanding, a theoretical model for the rapid computation of radiative properties for electrically small dimers of coupled SRRs with a single driven element was derived. This model was vital for identifying those dimer configurations (SRR orientations realtive to their line of centres) that produce superdirective emission. The model is quite generally applicable to consider dimers formed from SRRs of arbitrary size and operation at any frequency.

In particular, a key finding of this work is having reported that superdirectivity can occur only when the dimer is in the “CC” configuration, and having justified this result in physical terms based on the fact that only in this case both the in-plane electric and out-of-plane magnetic dipoles associated to each of the SRRs forming the dimer can be consistently driven to be almost in anti-phase as per requirements of superdirective arrays. The method is easily modified to study coupled resonators of any shape if numerical values are used for the current and charge distributions of a single element rather than relying on an analytical approximation for them. Furthermore, our approach can simplify the design of superdirective SRR arrays for a huge frequency range, possibly up to THz frequencies, where the model can be easily extended by incorporating in the equivalent circuit the kinetic inductance of the resonators and the additional loss effect due to boundary scattering (see^34^ and references therein).

Simulations and proof-of-principle experiments have been performed to validate the prediction of the new analytical model. Both numerical and experimental results have confirmed the potential for superdirective radiation of coupled SRR dimers. We have shown that the electrically small singly fed SRR dimer, where coupling provides the desired rapidly varying current distribution, exhibits endfire superdirectivity within < ~0.3 dB of the maximum theoretical equivalent dipole array value. The path is now open towards designing superdirective meta-arrays with a greater number of resonators and employing the dimer as the constituent element of larger highly directive linear and planar arrays with conventional feeding networks.

## Supplementary information

Supplementary Information 

## Data Availability

The research materials and data supporting this publication are available from the corresponding author on reasonable request.

## References

[1] Spyropoulos A, Raghavendra CS (2002). Energy efficient communications in ad hoc networks using directional antennas. Proceedings.Twenty-First Annu. Jt. Conf. IEEE Computer Commun. Societies.

[2] Mailloux, R. J. *Phased Array Antenna Handbook, Third Edition*. Artech House antennas and electromagnetics analysis library (Artech House Publishers, 2017).

[3] Hansen, R. C. *Electrically**Small, Superdirective, and Superconductive Antennas* (Wiley, 2006).

[4] Oseen CW (1922). Die Einsteinsche Nadelstichstrahlung und die Maxwellschen Gleichungen. Annalen der Phys..

[5] Bouwkamp CJ, de Bruijn NG (1946). The problem of optimum antenna current distribution. Philips Res. Rep..

[6] Uzkov AI (1946). An approach to the problem of optimum directive antenna design. Comptes Rendus (Doklady) de. l’ Academie des. Sci. de l’URSS.

[7] Balanis, C. A. *Antenna Theory: Analysis and Design*, 318, 4th edn (John Wiley & Sons, Hoboken, New Jersey, 2016).

[8] Uzsoky M, Solymar L (1956). Theory of superdirective linear arrays. Acta. Phys. Acad. Hung. Sci..

[9] Bloch A, Medhurst RG, Pool SD (1953). A new approach to the design of super-directive aerial arrays. Proc. IEE-Part III: Radio. Commun. Eng..

[10] Margetis D, Fikioris G, Myers JM, Wu TT (1998). Highly directive current distributions: General theory. Phys. Rev. E.

[11] Azevedo JAR (2011). Synthesis of planar arrays with elements in concentric rings. IEEE Trans. Antennas Propag..

[12] Shamonina E, Solymar L (2014). Maximum directivity of arbitrary dipole arrays. IET Microwaves, Antennas & Propag..

[13] Altshuler, E. E., O’Donnell, T. H. & Yaghjian, A. D. A superdirective array using very small genetic antennas. *Digest, URSI General Assembly, Maestricht* (2002).

[14] Altshuler EE, O’Donnell TH, Yaghjian AD, Best SR (2005). A monopole superdirective array. IEEE Trans. Antennas Propag..

[15] Buell K, Mosallaei H, Sarabandi K (2007). Metamaterial insulator enabled superdirective array. IEEE Trans. Antennas Propag..

[16] Kokkinos T, Feresidis AP (2012). Electrically small superdirective endfire arrays of metamaterial-inspired low-profile monopoles. IEEE Antennas Wirel. Propag. Lett..

[17] O’Donnell, T. H. & Yaghjian, A. D. Electrically small superdirective arrays using parasitic elements. In *Antennas and Propagation Society International Symposium 2006, IEEE*, 3111–3114 (IEEE, 2006).

[18] Yaghjian, A. D., O’Donnell, T. H., Altshuler, E. E. & Best, S. R. Electrically small supergain end-fire arrays. *Radio Science* **43** (2008).

[19] Sentucq, B., Sharaiha, A. & Collardey, S. Superdirective metamaterial-inspired electrically small antenna arrays. In *7th European Conference on Antennas and Propagation (EuCAP 2013)*, 151–155 (2013).

[20] Shamonina, E. & Solymar, L. Superdirectivity by virtue of coupling between meta-atoms. In *Advanced Electromagnetic Materials in Microwaves and Optics (METAMATERIALS), 2013 7th International Congress on*, 97–99 (IEEE, 2013).

[21] Pigeon, M., Sharaiha, A. & Collardey, S. Miniature and superdirective two elements endfire antenna array. In *8th European Conference on Antennas and Propagation (EuCAP 2014)*, 6–11 (2014).

[22] Shamonina, E. & Solymar, L. Superdirective “meta-molecules”. In *Advanced Electromagnetic Materials in Microwaves and Optics (METAMATERIALS), 2014 8th International Congress on*, 268–270 (IEEE, 2014).

[23] Haskou A, Sharaiha A, Collardey S (2015). Design of small parasitic loaded superdirective end-fire antenna arrays. IEEE Trans. Antennas Propag..

[24] Clemente A, Pigeon M, Rudant L, Delaveaud C (2015). Design of a super directive four-element compact antenna array using spherical wave expansion. IEEE Trans. Antennas Propag..

[25] Shamonina E, Kalinin VA, Ringhofer KH, Solymar L (2002). Magnetoinductive waves in one, two, and three dimensions. J. Appl. Phys..

[26] Solymar, L. & Shamonina, E. *Waves in Metamaterials* (Oxford University Press, 2009).

[27] Radkovskaya, A. *et al*. Experimental demonstration of superdirectivity for coupled dimers of meta-atoms. In *Advanced Electromagnetic Materials in Microwaves and Optics (METAMATERIALS), 2016 10th International Congress on*, 328–330 (IEEE, 2016).

[28] Radkovskaya A (2018). Superdirectivity from arrays of strongly coupled meta-atoms. J. Appl. Phys..

[29] Vallecchi, A., Li, L., Stevens, C. J. & Shamonina, E. Mapping directivity of coupled dimers of meta-atoms. In *2017 11th International Congress on Engineered Materials Platforms for Novel Wave Phenomena (Metamaterials)*, 358–360, 10.1109/MetaMaterials.2017.8107812 (2017).

[30] Vallecchi, A. *et al*. 3D printed superdirective dimer of split ring resonators. *IEEE Trans. Antennas Propag*. (manuscript submitted for publication).

[31] Liu N, Giessen H (2010). Coupling effects in optical metamaterials. Angew. Chem. Int. Ed..

[32] Chen, A., Kodigala, A., Lepetit, T. & Kanté, B. Multipoles of even/odd split-ring resonators. In *Photonics*, **2**, 883–892 (Multidisciplinary Digital Publishing Institute, 2015).

[33] Hesmer F (2007). Coupling mechanisms for split ring resonators: Theory and experiment. Phys. status solidi (b).

[34] Tatartschuk E, Gneiding N, Hesmer F, Radkovskaya A, Shamonina E (2012). Mapping inter-element coupling in metamaterials: Scaling down to infrared. J. Appl. Phys..

[35] Liu N, Liu H, Zhu S, Giessen H (2009). Stereometamaterials. Nat. Photonics.

[36] Powell DA, Lapine M, Gorkunov MV, Shadrivov IV, Kivshar YS (2010). Metamaterial tuning by manipulation of near-field interaction. Phys. Rev. B.

[37] Sersic I, Frimmer M, Verhagen E, Koenderink AF (2009). Electric and magnetic dipole coupling in near-infrared split-ring metamaterial arrays. Phys. Rev. Lett..

[38] Poo Y, Wu R-x, Liu M, Wang L (2014). A circuit model for the hybrid resonance modes of paired srr metamaterials. Opt. Express.

[39] Awai I, Zhang Y (2007). Coupling coefficient of resonators-an intuitive way of its understanding. Electron. Commun. Jpn. (Part. II: Electron.).

[40] Elnaggar SY, Tervo RJ, Mattar SM (2015). Energy coupled mode theory for electromagnetic resonators. IEEE Trans. Microw. Theory Tech..

[41] Elnaggar SY, Tervo RJ, Mattar SM (2015). General expressions and physical origin of the coupling coefficient of arbitrary tuned coupled electromagnetic resonators. J. Appl. Phys..

[42] Balanis, C. A. *Antenna Theory: Analysis and Design* (Wiley-Interscience, New York, NY, USA, 2016).

[43] Allen JE, Segre SE (1961). The electric field in single-turn and multi-sector coils. Il Nuovo Cimento (1955-1965).

[44] Sydoruk O, Tatartschuk E, Shamonina E, Solymar L (2009). Analytical formulation for the resonant frequency of split rings. J. Appl. Phys..

[45] CST Microwave Studio. User manual version 2016. CST Computer Simulation Technology GmbH, Darmstadt, Germany (2016).

[46] Vallecchi, A., Stevens, C. & Shamonina, E. Superdirective meta-arrays at telecommunication wavelengths. In *Loughborough Antennas Propagation Conference (LAPC 2017)*, 1–4, 10.1049/cp.2017.0283 (2017).

