# Supplementary material for: Superdirective dimers of coupled self-resonant split ring resonators: Analytical modelling and numerical and experimental validation
Source: Sci Rep. 2020 Jan 14;10:274. doi: 10.1038/s41598-019-56988-6 (PMC6959271; doi:10.1038/s41598-019-56988-6)
Supplement: Supplementary file 1 — Supplementary Information [file 41598_2019_56988_MOESM1_ESM.pdf]

Supplementary information

Article in *Scientific Reports*

Superdirective dimers of coupled self-resonant split ring resonators: Analytical modelling and numerical and experimental validation

A. Vallecchi[1,*], A. Radkovskaya[2], L. Li[1,3], G. Faulkner[1], C. J. Stevens[1], and E. Shamonina[2]
[1]Department of Engineering Science, University of Oxford, Parks Road, Oxford, OX1 3PJ, United Kingdom
[2]Magnetism Division, Faculty of Physics, M. V. Lomonosov Moscow State University, Leninskie Gory, Moscow 119992, Russia
[3]Insight Lifetech, Shenzhen, P.R. China, 518052
*Corresponding author: andrea.vallecchi@eng.ox.ac.uk

Supplementary Discussion S1

For a linear array of two parallel dipoles spaced by a distance d the directivity in the end-fire directions can be written as[1]

$$D = \frac{2 + \Re\left\{I_{01} I_{02}^{*} e^{\pm ikd}\right\}}{\left(|I_{01}|^2 + \right)\frac{2}{3} + 2p\Re\left\{I_{01} I_{02}^{*}\right\}} \tag{S.1}$$

where I_{0j}, $j = 1,2$ are the array excitation coefficients, k denotes the wavenumber, and the quantity p is given by (8) in the paper. For an ordinary end-fire phased array the excitation coefficients can be simply taken to be, for example, $I_{01} = 1$ and $I_{02} = e^{\mp ikd}$, which reduces (S.1) to

$$D^{pa} = \frac{3}{1 + \frac{3}{2}\cos^2(kd)\left\{\frac{1}{kd}\left[1 - \frac{1}{(kd)^2}\right]\tan(kd) + \frac{1}{(kd)^2}\right\}} \tag{S.2}$$

The optimal excitation coefficients for a superdirective two dipole array are shown in Eq. (7) of the paper and the corresponding maximum possible directivity in the end-fire directions is[1]

$$D^{sd} = I_1^{\text{opt}} + I_2^{\text{opt}} e^{\pm kd} \tag{S.3}$$

The directivity of end-fire superdirective and ordinary phased array dipole dimers calculated by (S.2) and (S.3) are compared in Fig. S1(a) at variable inter-element spacing d, while Fig. S1(b) shows the respective relative phases of the excitation coefficients for the two types of array. It can be noted that in the limit of vanishing distance between the dipoles, the directivity of the phased array reduces to that of a single element ($D = 1.5$), whereas the superdirective array reaches its upper theoretical limit of directivity $D^{sd} = 5.25$; this value is significantly higher than the maximum directivity of the phased array, $D^{pa} \cong 3.5$, but is obtained for out of phase excitation of the dipoles. As recalled in the paper, the realization of such a spatially fast varying current distribution is one of the most critical aspect of realizing a superdirective array.

Supplementary methods S2

Equivalent circuit for a pair of coupled SRR

By inspection of the equivalent circuit of a pair of electrically and magnetically coupled SRRs shown in Fig. 3 in the paper, the following linear system of equations can be written:

$$\begin{cases} V_1 = -\dfrac{1}{i\omega}\dfrac{C_2(I_0 - I_1) - KI_2}{C_1C_2 - K^2} = (R_1 - i\omega L_1)I_{1a} - i\omega M I_2 \\ V_2 = -\dfrac{1}{i\omega}\dfrac{C_1 I_2 - K(I_0 - I_1)}{C_1C_2 - K^2} = -(R_2 - i\omega L_2)I_2 + i\omega M I_{1a} \end{cases} \tag{S.4}$$

This system can be solved for the currents I_1 and I_2 in terms of I_0 (or equivalently in terms of V_1). As a result, equations (1) in the paper for I_1/I_0 and I_2/I_0 are obtained.

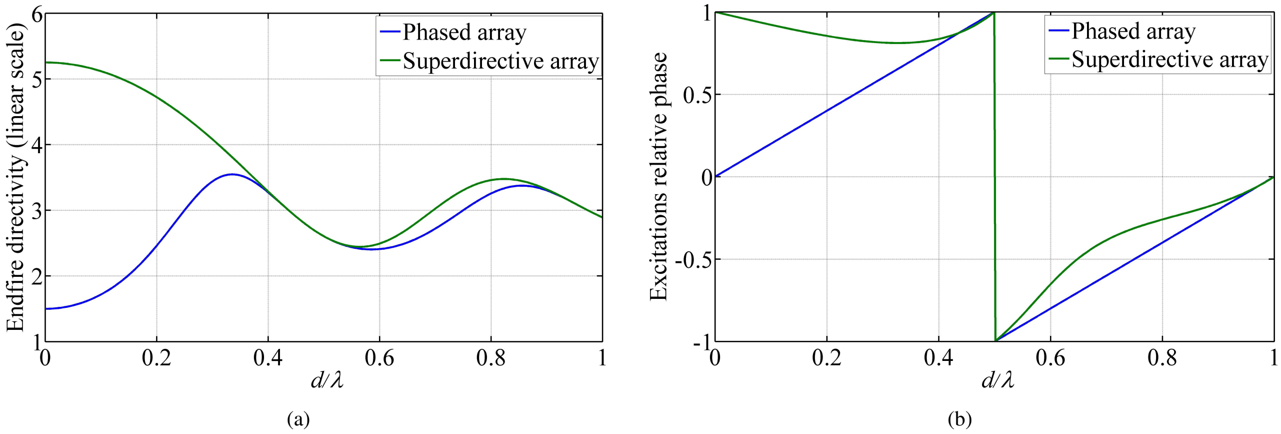

Supplementary Figure S1. Comparison of performance and prescribed element excitations for phased and superdirective arrays made of two elementary dipoles at variable inter-element spacing d: (a) directivity; (b) relative phase of the excitation coefficients.

Estimation of R, L, C parameters for a single SRR

In the equivalent circuit of the SRR dimer shown in Fig. 3 of the paper, R, L, and C represent the total resistance, self-inductance and self-capacitance of a single SRR. Different analytical expressions have been derived for the equivalent lumped element circuit of a single SRR in air[2–4]. While these formulas can predict with reasonable accuracy the SRR resonance frequency, they might be less accurate in separately estimating L and C, which in turn could undermine the overall effectiveness of our model. A numerical approach is instead adopted in this work and the unknown R, L, and C parameters are determined by curve fitting, with a least square criterion, both the input impedance and current functions that can be derived from the equivalent circuit of a single SRR to the corresponding data obtained with full-wave (FW) simulations. For a single SRR element the lumped circuit model simply consists of the primary side of the equivalent circuit of the SRR dimer with $M = K = 0$; therefore, the input impedance and current of the SRR can be expressed as

$$Z_i = \frac{(R - i\omega L)}{1 - i\omega C\,(R - i\omega L)}, \quad \frac{I_1}{I_0} = \frac{Z_i}{(R - i\omega L)} \tag{S.5}$$

In the simulations, performed with CST Microwave Studio (MWS), the reference values for the current have been calculated, based on Ampere's Law, as the circulation of the magnetic field at the section of the ring diametrically opposite to the gap, where for symmetry reasons the electric charge vanishes and the total current coincides with the conduction current.

For an SRR with outer diameter $D = 22$ mm, wall thickness $w = 0.8$ mm, wall height $h = 5$ mm, and gap width $g = 2$ mm, by using the above curve fitting method it is obtained $R = 8$ Ohm, $L = 21$ nH, $C = 0.3$ pF. Inserting these parameters into (S.5) provides the input impedance and current responses plotted in Fig. S2, where also the corresponding reference data simulated with CST MWS are reported for the purpose of comparison. As apparent, this approach, in spite of being computationally inexpensive, since it requires only one simulation of the single SRR, allows reproducing the ring response with good accuracy, including the position of the SRR resonance frequency.

Electric (K) and magnetic (M) coupling coefficients

The determination of the mutual capacitance, K, and mutual inductance, M, is accomplished by the same method used in[5]. This approach essentially consists of equating the field expressions of the electric and magnetic energies, including the effect of retardation, with the corresponding circuit quantities. The mutual magnetic and electric energies are calculated from the retarded vector and scalar potentials associated with the adopted normalised analytical current and charge distributions along the SRRs.

If the current density is replaced by a filament current, i.e., ignoring the variation of the current density across the cross section, the mutual magnetic energy can be expressed as

$$W_m^m = \frac{\mu_0}{4\pi} \int_{C_1} \int_{C_2} I_1(r_1) \cdot I_2(r_2) \frac{e^{ik|r_1 - r_2|}}{|r_1 - r_2|} dC_1 dC_2 \tag{S.6}$$

where C_1 and C_2 denote the circular paths going through the central points of the SRR cross sections, where currents are assumed to be concentrated, r_1 and r_2 are the position vectors of the infinitesimal current elements dC_1 and dC_2 (cf. Fig. 1(b)

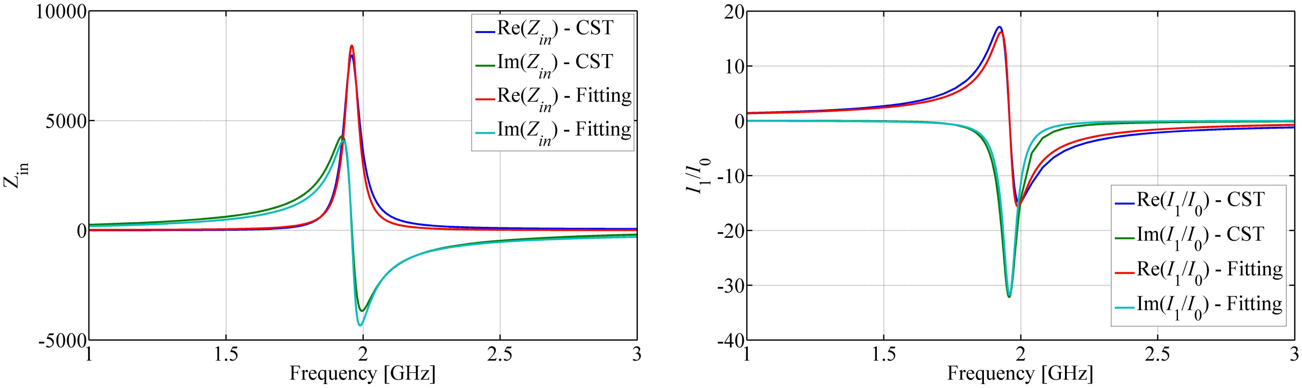

(a) (b)

Supplementary Figure S2. Real and imaginary parts of the (a) input impedance and (b) current in a single SRR with dimensions $D = 22$ mm, $g = 2$ mm, $h = 5$ mm, $w = 0.8$ mm. Results calculated with (S.5) and the parameters R, L, C obtained by curve fitting (red and cyan lines) are compared with the reference CST MWS simulated data (blue and green lines).

in the paper)on the two SRRs, and μ_0 is the free-space permeability. In terms of circuit quantities the mutual magnetic energy stored in two circuits instead reads as

$$W_m^m = MI_{1,t}I_{2,t} \tag{S.7}$$

where $I_{1,t}$, $I_{2,t}$ are the total currents that include the displacement current and are independent of space and conserved along the rings. Then, by equating (S.6) and (S.7), the mutual inductance can be derived as

$$M = \frac{\mu_0}{4\pi}\int_{C_1}\int_{C_2} I_{1,n}(r_1)\cdot I_{2,n}(r_2)\frac{e^{ik|r_1-r_2|}}{|r_1-r_2|}dC_1dC_2 \tag{S.8}$$

where $I_{j,n} = I_j/I_{j,t}$, $j = 1,2$ are normalised and dimensionless functions that describe the angular tapering of the current amplitude along the SRRs.

In an analogous manner, the mutual electric energy can be expressed in terms of the scalar potential and the charge densities as the following double integral

$$W_e^m = \frac{q_1q_2}{4\pi\varepsilon_0}\int_{C_1}\int_{C_2} \rho_{1,n}(r_1)\rho_{2,n}(r_2)\frac{e^{ik|r_1-r_2|}}{|r_1-r_2|}dC_1dC_2 \tag{S.9}$$

where $\rho_j(r_{j,n}) = \rho_j(r_j)/q_j$, with $j = 1,2$, are the linear charge densities on the SRRs normalised with respect to the corresponding total charges q_j on one half of each ring, between the gap and the SRR mid-section where the charge vanishes, and ε_0 is the free-space permittivity. It is noted that simplified versions of (S.6) and (S.9), disregarding the retardation term $e^{ik|r_1-r_2|}$, are commonly used[6,7]. In terms of circuit quantities[1] the mutual electric energy can be written as

$$W_e^m = -\frac{K}{(C^2-K^2)}q_1q_2 \tag{S.10}$$

Of the two solutions for K that (S.10) admits, the one that does not fulfil the limiting condition $K = 0$ for $W_e^m \to 0$ is discarded, and thus it is obtained

$$K = \frac{1-\sqrt{1+4C^2\left(W_{e,n}^m\right)^2}}{2W_{e,n}^m} \tag{S.11}$$

where $W_{e,n}^m = W_e^m/(q_1q_2)$. It is observed that, in the limit for $|\kappa_E| = |K/C| \ll 1$, (S.11) reduces to the approximate solution of (S.10)

$$K \cong -\frac{C^2}{q_1q_2}W_e^m \tag{S.12}$$

[1]The total charges on two coupled capacitors on the capacitors are connected to the applied voltages by the linear equations $Q_{1,2} = C_{1,2}V_{1,2} + KV_{2,1}$, or, equivalently, $V_{1,2} = \Delta^{-1}(C_{2,1}Q_{1,2} - KQ_{2,1})$, where $\Delta = C_1C_2 - K^2$. Then, the energy of two coupled capacitors can be written as $W_e = Q_1V_1/2 + Q_2V_2/2 = \Delta^{-1}\left(C_2Q_1^2/2 + C_1Q_2^2 - KQ_1Q_2/2\right)$ with the last term that contains the charges of both capacitors being the mutual energy[8,9].

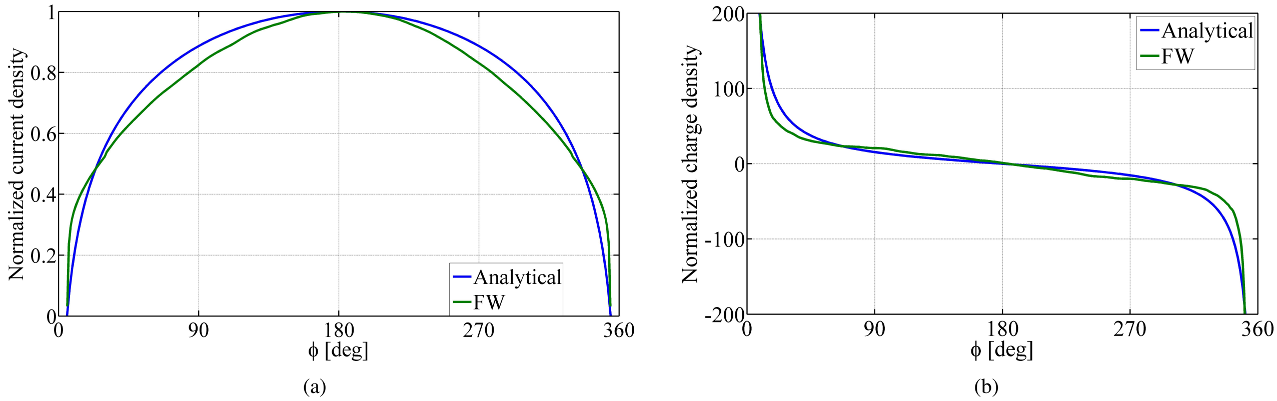

Supplementary Figure S3. (a) Normalised line current and (b) charge densities on an SRR: theoretical profiles employed in this work are compared with results of numerical simulation at the SRR resonance. SRR dimensions are: $r_0 = 11$ mm, $g = 2$ mm, $d = 5$ mm, $w = 0.8$ mm, $h = 5$ mm.

For the calculation of the integrals in (S.8) and (S.9) that give M and K, analytical approximations of the current and charge densities along the SRRs are used. For the current density we used the expression provided in[5] (see Eq. (16) in the paper). The normalised line charge density, which is connected to the filament current by the current continuity equation, reads as[5]

$$\rho_{j,n}(\phi;\phi_{g,j}) = \frac{\rho_j(\phi;\phi_{g,j})}{q_j} = -\frac{\cot\left[\dfrac{(\phi-\phi_{g,j})}{2}\right]}{2r_0 \ln\left[\sin\left(\dfrac{\delta}{2}\right)\right]}, \quad \delta+\phi_{g,j} \leqslant \phi \leqslant 2\pi-\delta+\phi_{g,j} \tag{S.13}$$

where all the parameters have the same meaning as in the paper (see Fig. 1). Moreover, the total charges on the SRRs can be obtained by integration of the charge densities (S.13) over the angular range $\delta+\phi_{g,j} \leqslant \phi \leqslant \pi+\phi_{g,j}$ and can be found to be

$$q_j = -\frac{2\varepsilon_0 V_j}{\pi}(h+w)\ln\left[\sin\left(\frac{\delta}{2}\right)\right], \quad j = 1,2 \tag{S.14}$$

where V_j are the voltage at the SRR gaps. The theoretical functions adopted for describing the normalized current and charge densities on an SRR are shown in Fig. S3 to provide reasonable approximations to the corresponding trends deduced from the numerical simulation of the electric and magnetic fields on the surface of an SRR at the resonance.

Coupled SRRs equivalent circuit validation

With all the parameters known, the equivalent circuit can be solved for the currents I_1 and I_2 in the fed and parasitically coupled SRRs, in terms of which any other circuit quantity can be obtained. The validity of the results provided by the equivalent circuit have been extensively tested by comparisons with the data obtained with FW simulations performed with CST MWS for sample dimer configurations with different orientations of the SRR elements. Besides the total currents in the SRRs, also the input impedance of the dimer has been verified, as it constitutes another important parameter that can give additional information on the accuracy of the equivalent circuit adopted in this work. The input impedance of the latter can be calculated as

$$Z_i = \frac{V_1}{I_0} = (R - i\omega L)\frac{I_1}{I_0} - i\omega M\frac{I_2}{I_0} \tag{S.15}$$

where the expressions of the currents ratios $I_{1,2}/I_0$ are given in (1) in the paper. In the limit of vanishing coupling $M = K = 0$, (S.15) reduces to the input impedance (S.5) of a single SRR.

For the purpose of illustration, a selection of results of the validation tests performed are presented relative to a few dimer configurations which are representative of distinct coupling regimes[5]. In all the examples, the SRRs have outer diameter $D = 22$ mm, wall thickness $w = 0.8$ mm, wall height $h = 5$ mm, and gap width $g = 2$ mm; the centre-to-centre distance is set to $d = 28$ mm, which corresponds to a separation distance of 6 mm. The reference values for the current have been obtained in the simulations by exploiting Ampere's Law, as described in the preceding section. The first dimer configuration shown is formed by two SRRs with the gaps facing each other, whose coupling is predominantly electric[5] and which is referred to as "near". The magnitude and phase of the ratio of the currents in the coupled SRRs of the "near" dimer are plotted against frequency

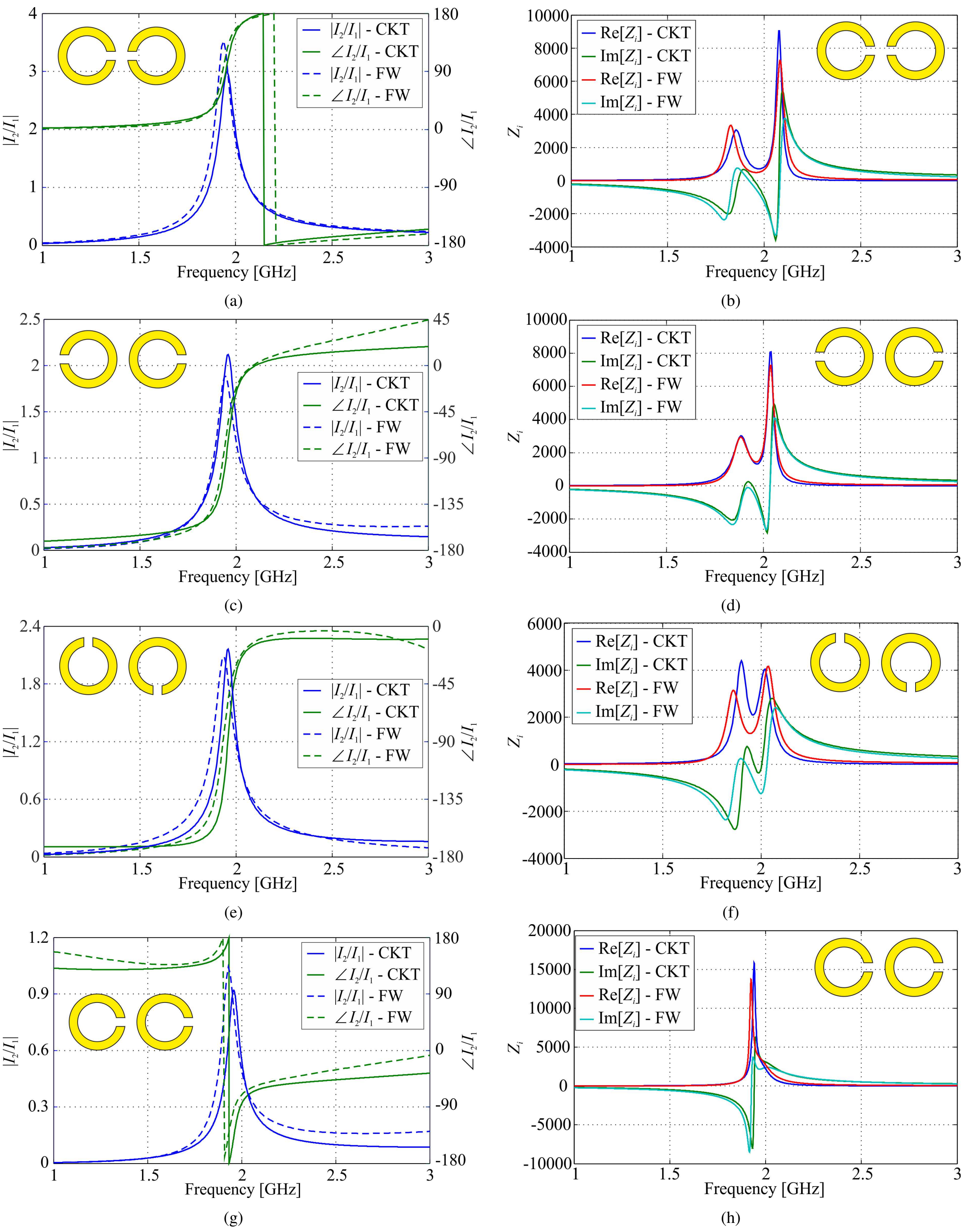

Supplementary Figure S4. Magnitude and phase of the ratio of the currents in the constituent SRRs of the dimers with the variable configurations shown in insets and real and imaginary parts of their input impedance. The dimer and SRRs dimensions are: $D = 22$ mm, $g = 2$ mm, $h = 5$ mm, $w = 0.8$ mm, and $d = 28$ mm. Currents and input mpedances of the dimers calculated with the equivalent circuit (CKT) in Fig. 3 in the paper are compared with corresponding FW simulation results. (a), (b) “Near”, (c), (d) “far”, (e), (f) “reversed”, and (g), (h) “CC” dimer structures.

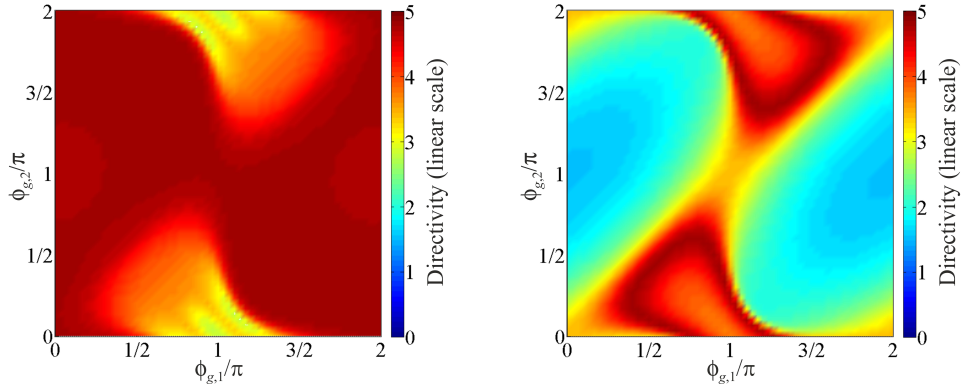

(a) (b)

Supplementary Figure S5. Maps of maximum directivity of an SRR dimer vs the angular orientations of its constituent elements for maximum radiation in the end-fire directions obtained by approximating each SRR as an open loop of uniform current: (a) $\theta = 90°, \phi = 180°$; (b) $\theta = 90°, \phi = 0°$. SRR dimensions are: $D = 22$ mm, $g = 2$ mm, $d = 5$ mm, $w = 0.8$ mm, $h = 5$ mm.

in Fig. S4(a) while the input impedance of the dimer is shown in Fig. S4(b). It can be observed that for all these quantities predictions by the equivalent circuit are in good agreement with results of FW simulations, especially in the region around the resonance frequency of the constituent SRR ($f_r \cong 1.96$ GHz). The dimer configuration in which the gaps lie at the opposite sides of the dimer axis ("far") is analysed in Figs. S4(c) and S4(d). Also for this arrangement, where coupling is dominated by its magnetic part[5], analytical results agree well with CST MWS simulations. The third and last examples reported are relative to the "reversed" and "CC" dimer structures, as shown in insets of Figs. S4(e), S4(f) and S4(g), S4(h). For these configurations the overall coupling between the SRRs has both electric and magnetic contributions which tend to reinforce or oppose each other[5]. As apparent, also in these cases the equivalent circuit is capable of reproducing with good accuracy both the ratio of the SRR currents and the input impedance of the dimer. These results confirm that the equivalent circuit can be used to generally describe the response of coupled SRRs with arbitrary orientation.

Supplementary results S3

Directivity of dimers of SRRs considered as open loops of uniform current

By applying the analytical model we have developed to sample the maximum directivity achievable in the SRR dimer configurational space, i.e. for different orientations of the SRRs, we show in the paper that optimum superdirectivity can only be produced by SRR dimers in the "CC" configuration or its reverse, when the SRR gaps are oriented in the same direction along the array axis. We also mention therein that incorporating in the model the tapering of the current density on the resonant SRRs is essential to correctly predict the radiation properties of an SRR dimer, and that, for example, approximating each SRR as an open loop of uniform current, which for small gaps would be practically equivalent to assimilate the SRR to a magnetic dipole, would completely impair the accuracy and reliability of the model. Here this point is further proved by presenting the maps of end-fire directivity in Fig. S5, which are constructed in the same manner as those in Fig. 6 of the paper and for the same pair of SRRs considered there, except that the currents circulating in the SRRs are assumed to be uniform. As anticipated in the paper, these maps appear to be substantially complementary to data in Fig. 5 which represent the degree of congruency of the excitation coefficients of the SRR dimers to the theoretical values for superdictivity. In fact, in the uniform current regime the element pattern has good circular symmetry in the plane of the rings and the actual dependency of the dimer radiation performance on the orientation of the SRRs is suppressed under this simplistic assumption.

Dimers of non-identical SRRs: Model applicability and trend of directivity

To demonstrate the existence of a unique optimal SRR dimer configuration meeting the requirements for superdirectivity, which is the main objective of this work, for simplicity the constituent elements have been considered to be identical. However, our model has no intrinsic limitation in this respect and it can be used to analyse dimers made of SRRs with different dimensions and to assess the trend of directivity for variable geometric characteristics of the dimer. For the purpose of illustration in the following we present the analysis of the effect on the dimer directivity of varying the inter-element distance, and the radius and gap width of one of the SRRs. We consider a dimer in the "CC" configuration with $\phi_{g,1} = \phi_{g,2} = \pi$, as sketched in the inset of Fig. S6(a). The dimensions of the fed SRR (shaded in red in the figure) are fixed ($r_1 = 11$ mm, $g_1 = 2$ mm, $h_1 = 5$ mm,

$w_1 = 0.8$ mm), as well as the height and conductor thickness of the parasitically coupled one ($h_2 = h_1$ and $w_2 = w_1$). In Fig. S6 the directivity in the backfire direction $\theta = 90°, \phi = 180°$ calculated against frequency with the analytical model is compared with the corresponding FW simulation results.

in Figs. S6(a) and S6(b) it can be observed that the model and simulations similarly predicts that there exists an optimal distance at which the directivity of the dimer reaches its maximum; in simulations the spread in frequency of the dimer directivity responses is slightly more accentuated, especially for smaller inter-element distances; this might be due to the fact that in the model the adopted profile for the current along the rings is invariant, while in practice there could be some alteration and deformation of the current distributions, especially when the SRRs are very close to each other, which in turn can reflect into a stronger coupling and wider split of the coupled resonance frequencies.

Furthermore, similar trends of the dimer directivity are found with the model snd simulations when the radius or the gap of one of the SRRs is variable, which is shown in Figs. S6(c), S6(d) and Figs. S6(e), S6(f), respectively. In particular, a larger radius of the parasitic SRR produces a higher directivity, even though this does not happen as a result of a more precise fulfillment of the superdirecitivity conditions for the currents , but essentially just because of the larger size of one of the radiating element. In this connection, it is worth noting that the lower dimer directivity that the model generally estimates with respect to simulations is simply due to replacing the actual SRRs with filament currents that we localize at the centre of the SRR cross sections rather than on their outer surfaces (cf. Fig. 1 in the paper).

In conclusion, these results support the applicability of the model also in the case of non-identical SRRs: in spite of the simplifying assumptions introduced with the aim of achieving a numerically efficient implementation, the model is satisfactorily accurate in predicting the trend of directivity when the geometric characteristics of the dimer are varied, and it can thus provide useful indications for the practical design of a superdirective SRR dimer.

It is worth noting that selecting different SRR radii and/or different widths of the gaps would provide additional degree of freedom to possibly achieve values of directivity closer to the theoretical maximum, and to facilitate impedance matching to the antenna feed, as required in a practice.

References

1. Altshuler, E. E., O'Donnell, T. H., Yaghjian, A. D. & Best, S. R. A monopole superdirective array. *IEEE Trans. Antennas Propag.* **53**, 2653–2661 (2005).

2. Zhou, L. & Chui, S. T. Eigenmodes of metallic ring systems: A rigorous approach. *Phys. Rev. B* **74**, 035419 (2006).

3. Du, J., Liu, S., Lin, Z. & Chui, S. T. Magnetic resonance of slotted circular cylinder resonators. *J. Appl. Phys.* **104**, 014907 (2008).

4. Sydoruk, O., Tatartschuk, E., Shamonina, E. & Solymar, L. Analytical formulation for the resonant frequency of split rings. *J. Appl. Phys.* **105**, 014903 (2009).

5. Tatartschuk, E., Gneiding, N., Hesmer, F., Radkovskaya, A. & Shamonina, E. Mapping inter-element coupling in metamaterials: Scaling down to infrared. *J. Appl. Phys.* **111**, 094904 (2012).

6. Landau, L. D. & Lifshitz, E. M. *Electrodynamics of Continuous Media* (Pergamon, Oxford, 1984).

7. Simonyi, K. *Foundations of Electrical Engineering: Fields—Networks—Waves* (Pergamon, Oxford, 1963).

8. Smythe, W. *Static and Dynamic Electricity* (1989).

9. Whitaker, J. C. *The Electronics Handbook* (Crc Press, 2005).

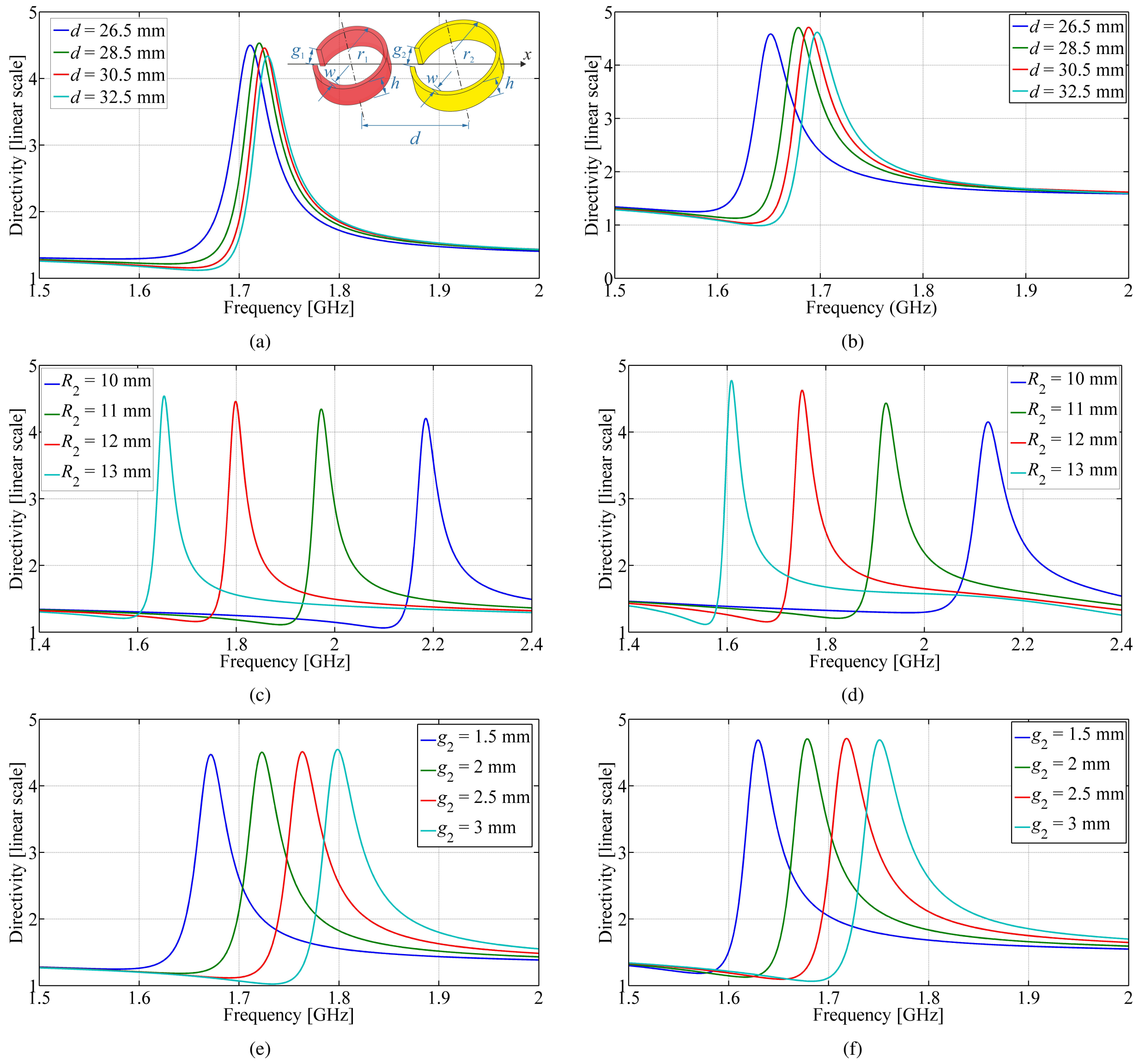

Supplementary Figure S6. Directivity of dimers of non-identical SRRs. The dimensions of the fed SRR are fixed $D_1 = 22$ mm, $g_1 = 2$ mm, $h_1 = 5$ mm, $w_1 = 0.8$ mm. Moreover, $h_2 = h_1$ and $w_2 = w_1$. Results calculated with the analytical model are compared with the corresponding FW simulation results. (a), (b) variable distance between the SRRs, $d = 3, 5, 7, 9$ mm, with $R_2 = 12.5mm$ and $g_2 = 2$ mm; (c), (d) variable $R_2 = 10, 11, 12, 13$ mm, with $d = 5mm$ and $g_2 = 2$ mm; (e), (f) variable $g_2 = 1.5, 2, 2.5, 3$ mm, with $R_2 = 12.5mm$ and $d = 5$ mm.
